# Religiosity, school connectedness, and tobacco use susceptibility: a longitudinal study of adolescents in Mumbai and Kolkata, India

**DOI:** 10.1186/s12889-025-25035-7

**Published:** 2025-11-07

**Authors:** William J. McCarthy, Ritesh Mistry, Namrata K. Puntambekar, Trivellore Raghunathan, Michael J. Kleinsasser, Maruti B. Desai, Prakash C. Gupta, Mangesh S. Pednekar

**Affiliations:** 1 Fielding School of Public Health, University of California-Los Angeles, A2-125 CHS, mc 690015; 650 Charles Young Drive;, Los Angeles, CA 90095-6900 United States of America; 2 Center for Cancer Prevention & Control Research, University of California-Los Angeles, Los Angeles, CA United States of America; 3 Department of Psychology, University of California-Los Angeles, Los Angeles, CA United States of America; 4 Department of Health Behavior and Health Education, University of Michigan, Ann Arbor, MI United States of America; 5 Healis Sekhsaria Institute for Public Health, Mumbai, India; 6 Survey Research Center, Institute for Social Research, University of Michigan, Ann Arbor, MI United States of America; 7 Department of Biostatistics, University of Michigan, Ann Arbor, MI United States of America

**Keywords:** Prosocial factors, Hindu, Muslim, Adolescence, Tobacco use susceptibility, India, Religiosity, School connectedness, Mumbai, Kolkata

## Abstract

**Background:**

Religiosity and school connectedness have been shown to protect adolescents from tobacco use initiation in the U.S. and Europe but have not been examined in India. A population-based in-home survey of 1,982 adolescents’ susceptibility to tobacco use in India was examined in relation to several adolescent prosocial factors: connectedness with school, and three indicators of religiosity.

**Methods:**

Religiosity measures included participant frequency of attendance at places of worship (e.g., mosque, temple), frequency of prayer, and importance of prayer. School connectedness measures included feeling like you are a part of the school, you are happy at your school, and you feel safe at your school. Primary outcome was susceptibility to tobacco use defined as intention to or openness to using tobacco during next 12 months.

**Results:**

More in Mumbai than in Kolkata, adolescent prosocial factors were associated with reduced susceptibility to tobacco use. Adolescents’ sex-at-birth also influenced these associations.

**Conclusion:**

Encouraging religiosity and school connectedness may help reduce adolescent susceptibility to tobacco use in India.

**Supplementary Information:**

The online version contains supplementary material available at10.1186/s12889-025-25035-7.

Adolescent uptake of tobacco use is associated with near-term and long-term negative health consequences [1, 2]. Most adult tobacco users began as adolescents or emerging adults [3]. Evaluating risk of tobacco use in adolescent never smokers requires probing them about their future susceptibility to tobacco use. Susceptibility to smoking tobacco has been assessed in adolescents with no previous tobacco use (i.e., “ tobacco-naive users”) by asking, “Do you think you will be smoking cigarettes one-year from now [4] or Would you smoke a cigarette if a friend offered it to you [5, 6]?” Similar questions have assessed child susceptibility to using other tobacco products [7, 8]. Susceptibility to smoking, defined as the absence of a firm decision not to smoke, was a stronger predictor of subsequent smoking initiation than such well-established risk factors as the presence of smokers in the family or the smoking status of the child’s best friends [9].

Adolescence is the developmental period of greatest risk of tobacco use onset as the risk of tobacco use onset drops precipitously once adulthood has been reached [2]. Worldwide government efforts to reduce adolescent tobacco use initiation have had some success [10] but many adolescents with no previous tobacco use experience nevertheless remain susceptible to tobacco use initiation through emerging adulthood [11]. A 2012–2015 representative tobacco use survey of Indian respondents aged 15–24 years documented 5% who smoked cigarettes and 10.5% who used smokeless tobacco [3], rates comparable to youth rates of tobacco use in many other countries [12]. A subsequent (2019) state-specific adolescent tobacco use survey showed that 5.8% of boys and 4.4% of girls ages 13–15 years reported currently using tobacco products (combustible + smokeless) in Maharashtra [5]. The percentages of never cigarette smokers among these adolescents who reported susceptibility to future tobacco use were 8.8% of the boys and 6.4% of the girls [5]. The same survey administered in West Bengal showed that 11.5% of boys and 3.6% of girls ages 13–15 years reported currently using tobacco products [6]. The percentages of never cigarette smokers among these West Bengal adolescents who reported susceptibility to future tobacco use were 8.1% of the boys and 4.9% of the girls [6].

The consistently higher rates of tobacco use in male tobacco use susceptibility observed in Maharashtra and West Bengal reflect consistent differences by sex-at-birth in tobacco use observed throughout India [13–15], in other Asian countries [16, 17] and in other low-income and middle-income countries [18]. Differences in rates of tobacco use by sex-at-birth have dropped in high-income countries but remain high in low- and middle-income countries [19]. Smoking continues to be socially stigmatized among women in low- and middle-income countries, leading to under-reporting of smoking by women but also a higher prevalence of smokeless tobacco use among women [19] and higher rates of smoking and smokeless tobacco use by men [19].

Research on adolescent tobacco use initiation in India has traditionally emphasized risk factors, such as peer tobacco use [20], familial risks [20, 21], low socioeconomic status [3, 22, 23], and accessibility to tobacco products [20, 24]. These are the factors that have been studied the most due to their demonstrated impact on adolescent susceptibility to tobacco use initiation.

However, there has been a growing recognition outside of India of the importance of investigating health-promotive and protective factors that might mitigate risks associated with adolescent tobacco use initiation [25–27]. They refer to conditions or attributes of adolescents and their environment that reduce their likelihood of engaging in harmful behaviors. Examples in the context of tobacco use initiation include school connectedness [26, 28], religiosity [29–31], parental monitoring [30, 32], and community support systems [29].

School connectedness refers to how much students feel engaged, supported, and valued when attending school. The available evidence from studies outside of India suggests that adolescents who feel a high degree of connection to their schools are less likely to engage in risky behaviors such as tobacco use [25, 26, 33]. Similarly, religiosity, which encompasses religious beliefs, practices, and involvement in religious communities, has been associated with lower rates of substance use among adolescents outside of India [29, 30, 34, 35]. Given the pervasive influence of religion in Indians’ daily lives [36], examining the potential protective effects of religiosity on tobacco use susceptibility in adolescents is understudied in the Indian context.

The multifaceted nature of adolescent tobacco use initiation in India suggests that exploring both risk and protective factors would be essential for developing culturally appropriate, comprehensive prevention strategies. Increased understanding of the interplay between these factors in the Indian context should yield more effective interventions designed to reduce the prevalence of tobacco use among adolescents in India.

It is well established in Problem Behavior Theory (PBT) [37, 38] and Resiliency Theory [39] that adolescents who engage in prosocial behaviors, such as active involvement in religious activities and community service, are less likely to use tobacco compared to their peers who do not engage in such behaviors [40, 41]. In contrast to Zimmerman’s Resiliency Theory, PBT acknowledges prevention benefit from protective factors but has historically focused more on problem behaviors that predict tobacco use whereas Resiliency Theory focuses more on bolstering protective factors that can prevent adolescents from initiating tobacco use. Adolescents’ prosocial behaviors and attitudes are also inversely related to their susceptibility to tobacco use [42, 43], among other forms of drug use [37]. These prosocial factors include having a positive orientation to school (e.g., school connectedness) and regular attendance at a place of worship [31, 37]. Indeed, school connectedness [25, 44–48] has consistently been associated with reduced adolescent tobacco use [49].

Another major source of non-family socialization during childhood and adolescence is involvement in religious activities [50], which has been associated with reduced risk of adolescent cigarette smoking [41, 51, 52]. In the U.S., adolescent smoking has been inversely associated with frequency of attendance at a place of worship [51–53]. Religiosity measured in multiple ways among wave 1 nonsmokers in the (U.S.) National Longitudinal Study of Youth showed consistent inverse associations with risk of smoking initiation [54]. Most previous studies of religiosity and adolescent and young adult tobacco use have occurred in western countries where most religious affiliations are Christian [53–59]. We identified one study of religiosity as a predictor of tobacco use in a Muslim-dominated country, namely Indonesia [60], where Muslims who prayed regularly were less likely to smoke.

In India, where the predominant religions are Hinduism and Islam, it is unclear whether commonly used measures of religiosity are associated with tobacco use risk in youth. 84% of adult Indians report that religion is important in their lives and 60% report praying daily [36]. This strong support for religion is found across all major religious affiliations in India, found in rural and urban areas and found in people varying widely in educational attainment [36].

Religious affiliation but not religiosity has been assessed in adolescents in India, showing that tobacco use did not differ between Hindu and Muslim students in New Delhi [61]. Among adults in India, however, data showed that Muslims, Christians, and Buddhists used tobacco more than Hindus [62, 63]. Muslim antipathy to smoking may be ascribed to recent fatwas and Islamic legal opinions condemning tobacco use [64]. As evidence accumulated that tobacco use contributes to avoidable disease, Islamic scholars have increasingly cited Koranic injunctions against harming the human body as the basis for interpreting Islamic law as either discouraging (makruh) or prohibiting (haram) tobacco use [64, 65]. For many Hindus, tobacco use is seen as interfering with self-realization and is hence, discouraged [66]. Whether negative associations of religiosity with tobacco use susceptibility would be observed in Hindu and Muslim adolescents in India for either smoking or smokeless tobacco use is unknown.

Gender differences have been observed consistently and worldwide for tobacco use behaviors [67–69], for engagement with their schools [70, 71], for adherence to established behavioral norms [72], and for religiosity [30, 73]. Hence, all expected associations included interactions with sex-at-birth.

Given this high level of religiosity in India, and given previous evidence that adolescent religiosity is associated with reduced risk of tobacco use initiation in other countries [74–76], we tested the hypothesis of a prosocial influence of religiosity on tobacco use susceptibility in youth with no previous tobacco use experience. To address this and other important gaps, a four-year, longitudinal household survey of adolescent tobacco use was launched in 2018 in two large, culturally and geographically disparate Indian cities, Mumbai and Kolkata, in order to assess a variety of hypotheses concerning Indian early adolescent tobacco use initiation and possible environmental influences, including adolescents’ religiosity and school connectedness. In brief, one goal of the present study was to evaluate whether the sex-specific prosocial factors of engagement with school and three indicators of religiosity in Indian adolescents are associated with Indian adolescents’ susceptibility to tobacco use, as predicted by Problem Behavior Theory [37, 38] and Resiliency Theory [39]. Another goal was to document whether the inverse associations previously observed in western populations between these prosocial factors and tobacco use are also observed in Hindu and Muslim adolescents drawn from two geographically, linguistically and culturally distinct urban centers in India, Mumbai and Kolkata. Adolescents’ retail access to tobacco products in India’s major urban centers is high because of these cities’ high tobacco retailer density and low enforcement of India’s Cigarette and Other Tobacco Products Act [77].

## Methods

### Study design, participants and setting

In 2018–2019 (Wave 1 (W1)), 1,982 households with a 12-, 13-, or 14-year-old adolescent were randomly sampled from 52 communities in Mumbai and Kolkata to complete in-home questionnaires from face-to-face interviews about tobacco use. In 2019−20 (Wave 2 (W2)), 1,756 of these households participated in a one-year follow-up survey. This multi-stage, stratified, random sample was designed to be representative of neighborhoods and households with age-eligible adolescents in both cities by using the Urban Frame Survey (NSSO, 2017) sampling frame from the National Sample Survey Organization (NSSO) [78] of the Indian Ministry of Statistics and Programme Implementation (Mistry et al., 2018). The primary sampling unit was the Investigator Unit, a well-defined and clearly demarcated geographical area consisting of about 20 to 40 blocks. Each block has 120–150 households [78]. The stratification controlled for socioeconomic status. Within each selected primary sampling unit (stage 1), a socioeconomic status-stratified random sample of blocks (stage 2) was taken. Within each sampled block, all households were visited and evaluated for eligibility, and then recruited. The response rates of eligible households were 93% in Kolkata and 86% in Mumbai. When households included more than one adolescent of eligible age, the adolescent with the next birthday closest to the interview date was selected.

These data comprised the first two waves of a 4-wave longitudinal study of adolescent tobacco use in Mumbai and Kolkata [79]. For each eligible adolescent participant, one parent/guardian defined as caregiver was also interviewed at each assessment. The initial cohort included only 12, 13, and 14 year olds at the time of recruitment, with the expectation that a small but significant proportion would become tobacco users in the ensuing three years [61].

For the W1 data, no variable in the analyses exceeded 1.4% of observations being missing. For the W2 data, the 226 cases (11.4% attrition) lost to follow-up were taken into account by the W2 sampling weights. Within the W2 data, no variable in the analyses exceeded 1.9% of observations being missing.

#### Mumbai

 Mumbai (2023 population estimate = 27,180,000) [80] is the 2nd most populous city in India. An estimated 16.8% of Maharashtra’s population lives in Mumbai, the state’s biggest city [81, 82].

Mumbai is the financial, commercial and entertainment capital of India [83] and one of the world’s top ten centers of commerce [84]. Its residents exhibit a great diversity of languages and religious preferences [83]. Mumbai’s most dominant religious affiliations are Hindu (66%), Muslim (20.6%), Buddhist (4.8%), Jain (4.1%), Christian (3.3%), Sikh (0.5%), and Other (0.7%) [85].

#### Kolkata

 Kolkata is the 3rd most populous city in India (2023 population estimate = 15,500,000) [86, 87]. An estimated 15% of West Bengal’s residents live in Kolkata, also the state’s biggest city [88, 89]. Kolkata is the historical cultural capital of India and is the principal commercial, cultural, and educational center of East India [90, 91]. In recent decades, the growth of Kolkata’s economy has lagged that of other major Indian cities and attracted fewer international investors [92]. The foreign-born population of Kolkata dwindled since 1947, resulting in religious affiliations being limited mostly to Hindu (78%) and Muslim (20%) with a small number associated with Christian (0.9%) and Jain (0.5%) [93].

#### Translations

 The surveys were developed in English and translated into Hindi, Marathi and Bengali. A native speaker of the target language translated the English text into the target language. A second translator fluent in English and the target language then back-translated the survey from the target language back to English. Discrepancies between the original English text and the back-translated English text were resolved through cognitive testing with community members fluent in the target language.

### Study variables

Sensitive information such as the adolescent’s tobacco use behaviors was collected using an audio computer-aided self-interviewing system (Open Data Kit (ODK)) to ensure confidentiality. The English version of the baseline adolescent respondent questionnaire with all variables described below is included in online supplemental file #1.

#### Sociodemographic variables

 Standard sociodemographic measures were obtained from the participants (e.g., age, sex-at-birth, language preference, parent educational attainment) as well as questions about their religious affiliation in W1. For comparability to previous research on youth tobacco use susceptibility [94], W1 sex-at-birth and adolescent age were included as covariates in all regression analyses.

Parents’/caregivers’ top academic attainment was assessed by the following questions: “What is the highest level of education you have finished?” and “What is the highest level of education your spouse has completed?” Answer options were: No formal education, up to primary school (up to class IV), middle school class (V to VII), secondary school (ITI course, class VIII/X, or intermediate), College graduate (BA, BSc, Diploma, etc.), Postgraduate/professional degree, Above postgraduate degree (i.e., Ph.D.). The highest educational level reported for parent/guardian or spouse was used. To normalize the skewed distribution, this variable was recategorized as “Less than primary school,” “Completed primary school,” “Completed middle school,” “Completed secondary school,” and “Completed college or more.”

#### Religious affiliation

 Religious affiliation was documented by having the participant choose one of the following in response to the question, “What is your religion?“: “1) Hindu,” “2) Muslim,” “3) Christian,” “4) Sikh,” “5) Buddhist,” “6) Jain,” “7) Other: (specify).”

#### Preferred language of interview

 Participants could choose one of three languages to use during the interview, English, Hindi or the dominant regional language in each city. In Mumbai, the dominant regional language was Marathi; in Kolkata it was Bengali.

#### Type of school

 Primary and secondary schools in India are commonly classified into three categories: government-run, private and government-aided. Government-run schools are fully owned, operated and managed by either the state or national government. Private schools are owned, operated and managed by private individuals, organizations or trusts. They operate independently of government control. Government-aided schools have characteristics of both private and government-run schools as they are privately managed (often by religious organizations) but rely on government funding for key areas. Their curricula typically follow the state or central government ‘s curricula. Government-run schools aim to provide accessible education to all, regardless of income. Private schools cater to wealthy families and government-aided schools charge fees that are lower than private school fees, permitting them to appeal to middle-income families.

#### Tobacco use-related measures

 The primary outcome variable, *susceptibility to smoking* was assessed as “At any time during the next 12 months, do you think you will smoke a cigarette or bidi?” Another primary outcome variable, *susceptibility to using smokeless tobacco*, was assessed as “At any time during the next 12 months, do you think you will chew or apply tobacco in any form?” Answer options were: “Definitely not,” “Probably not,” “Probably yes,” “Definitely yes.” These questions and answer options have been used repeatedly in the India version of the Global Youth Tobacco Survey (administered 4 times in India) [95, 96] and in the authors’ earlier adolescent tobacco survey work [97]. For comparability to previous literature on susceptibility to tobacco use in young adolescent never users [9, 94, 98], these responses were dichotomized, with any answer other than “Definitely not” seen as evidence of increased adolescent openness to using tobacco within the next year. For evaluating susceptibility across W1 and W2, a consistent answer of “Definitely not” in both W1 and W2 was coded zero and all other possible answers were coded as “Maybe” susceptible.

#### Ever use of tobacco

 To screen out adolescents already experienced in tobacco use, a question about ever past use of tobacco was also included: “Have you ever tried or experimented with any form of tobacco, even once or twice?” Answer options were: “Never,” “Once or twice,” “Three or more times.” An answer of “Not sure” or “Don’t know” or refusal to answer this question was treated as evidence of ever having used, to minimize the possibility of adolescent under-reporting of tobacco use. This question was adapted from similar questions on the National Youth Tobacco Survey [99].

#### School connectedness measures

 The three school connectedness items used in this study were taken from the National Longitudinal Study of Adolescent Health, which showed that school connectedness was negatively associated with cigarette smoking [100]. The adolescent respondent was asked the question, “Do you agree or disagree with the following statements? 1) “You feel like you are a part of the school,” 2) “You are happy to be at school,” and 3) “You feel safe in your school.” Answer options were: Agree, Neither Agree Nor Disagree, Disagree. In analyses, these three measures were reverse-scored and totaled, so that a high score was associated with higher perceived school connectedness. To normalize a skewed distribution, this measure was dichotomized, with either a "Neither Agree Nor Disagree" or "Disagree" answer to any of the three indicators of school connectedness yielding a zero value on the composite measure of school connectedness.

#### Religiosity measures

 The adolescent participants answered three questions about their religiosity at wave 1, taken from the largest ever U.S. survey of religious observance by the Pew Research Center [101]. The first two questions assessed personal religiosity without distinguishing whether the participant’s motivation for engaging in prayer were intrinsic or extrinsic [102]; the third question assessed organizational religiosity, an objectively observable measure that has been widely used in studies of religiosity and health [102, 103].

##### Non-organizational religiosity

The first religiosity question was “How often do you pray?” Answer options were: “Never,” “1–3 times/year,” “1–3 times/month,” “1–3 times/week,” “Nearly every day.” To normalize a skewed distribution this variable was recategorized, collapsing the first three categories into “1–3 times/month or less.”

##### Importance of religiosity

The second religiosity question was “How important is prayer to you?” Answer options were: “Not at all important,” “Somewhat important,” “Very important.”

##### Organizational religiosity

The third religiosity question concerned the frequency of attendance at a place of worship, arguably the most common indicator of religiosity used in research: “How often do you go to a place of worship? (e.g., temple, mosque, church)” Answer options were: “Never,” “1–3 times a year,” “1–3 times a month,” “1–3 times per week,” and “Nearly every day.”

This study was reviewed and approved by the institutional review boards at the University of Michigan, University of California-Los Angeles and Healis Sekhsaria Institute for Public Health. Adolescent participation occurred only if both adolescent assent and parent written consent had been obtained in Wave 1 and informed consent in Wave 2.

### Statistical analysis

To ensure that outcomes were representative of the populations being studied, data were analyzed separately for Mumbai and Kolkata. The Stata version 18 suite of survey commands (Stata Corp, College Station, TX) was used to enable use of stage 1 and stage 2 sampling weights [79] to reflect the survey design, to correct for variation in non-response and to ensure that results were representative of the youth population in each city. The subpopulation option of Stata’s survey commands was used to analyze subgroups of participants (e.g., never-tobacco-users) while preserving the sample standard errors. Hypotheses were evaluated first with wave 1 cross-sectional data, and second with longitudinal data using wave 1 religiosity measures to predict change in one-year follow-up susceptibility to overall tobacco use. Chi square tests were applied to within-wave 1 (W1) data to compare the distributions of demographic characteristics of participants in Mumbai and Kolkata. Most within-wave 1 regression analyses consisted of logistic regression, in keeping with past research practice where tobacco use susceptibility among young adolescents was dichotomized prior to analysis [9, 94, 104]. Cross-wave analyses involved W1 predictors and W1-W2 change scores, with W2 sampling weights used to control for non-response. Susceptibility outcomes were marginal probabilities (expressed as percentages of Wave 1 tobacco-naïve participants), varying from 0.0 to 1.0, resulting from application of Stata’s margins command following output from Stata’s logistic regression program. Consistent with past practice [9, 98], hypotheses were tested on W1 tobacco-never-users only. Consistent with past scientific literature, analyses were stratified by sex at birth.

To optimize statistical power, the focal outcome reported here was susceptibility to any tobacco product (combining susceptibility to future smoking and smokeless tobacco use). The phi coefficient summarizing the association between the binary values for participants’ susceptibility to using smokeless tobacco and using smoking tobacco was calculated to confirm that the two outcomes were strongly associated. For readers wanting smoking-specific and smokeless tobacco use-specific results, they are reported in supplemental online file #2. More general regression statistics pertinent to the marginal means-based results reported in text are in supplemental online file #3.

#### Propensity score matching analyses

 Propensity score matching (PSM) is a statistical technique used to reduce bias and make two groups statistically more comparable in observational studies [105]. In view of the differences in their respective demographic characteristics, Mumbai and Kolkata mean impacts of religiosity and school connectedness on adolescent tobacco use susceptibility were compared after implementation of propensity score matching to make the two cities more analytically comparable and to reduce the effect of major confounders such as differences in parent educational attainment between the two cities. For these analyses, the matching variables included adolescent sex-at-birth, adolescent language preference, adolescent age, adolescent religious affiliation and parents’/guardians’ highest educational attainment.

#### Covariates

 For all regression analyses, covariates included adolescent age and sex-at-birth because these demographic characteristics have been associated consistently with susceptibility to tobacco use [106–109].

## Results

Table 1 describes the weighted W1 percentages of participants for selected demographic characteristics, stratified by city of residence. The distributions by sex, age and parent top educational attainment were generally comparable between Mumbai and Kolkata, with a few exceptions. The 9.5% (95%Confidence Interval (CI): 6.7, 13.3) of Kolkata parent/guardian participants reporting no formal education was higher than the 3.0% (CI: 1.5, 5.6) reported in Mumbai. While Hindus and Muslims comprised more than 90% of the religiously affiliated in both cities, 8.9% (CI: 5.4, 14.4) of households in Mumbai were affiliated with other religions (e.g., Jains, Christians, Buddhists, etc.), whereas the corresponding percentage for Kolkata was only 0.7% (CI: 0.3, 1.9). The distribution of adolescent choice of language to use during the interview differed between the two cities (F(1.68, 75.5) = 38.1, *p* <.001). Only 34% (CI: 26.0, 43.0) of adolescents in Mumbai selected the regional language (Marathi) compared to 83% (CI: 74.1, 88.8) of adolescents in Kolkata, who chose the regional language (Bengali). The corresponding percentages who chose the national language, Hindi, were 58.2% (CI: 49.1, 66.8) in Mumbai and 8.1% (CI: 5.2, 12.5) in Kolkata.

**Table 1:** Wave 1 sociodemographic characteristics of all participants(weighted), stratified by city and sex at birth, showing prevalence estimates and associated 95% confidence intervals

Demographic measures		Mumbai *n*^a^ = 481*	Mumbai *n*^a^ = 462	Mumbai *N*^a^ = 943	Kolkata *n*^b^ = 508	Kolkata *n*^b^ = 530	Kolkata *N*^b^ = 1038
		Males	Females	Total	Males	Females	Total
		weighted % (95% CI)	weighted % (95% CI)	weighted % (95% CI)	weighted % (95% CI)	weighted % (95% CI)	weighted % (95% CI)
Age at recruitment	12 years	33.1% (26.5, 40.5)	39.1% (33.3, 45.2)	36.1% (31.5, 41.0)	34.6% (30.3, 39.1)	36.8% (28.5, 46.0)	35.8% (30.9, 40.9)
	13 years	36.8% (28.7, 45.7)	37.1% (31.1, 43.6)	37.0% (32.6, 41.6)	40.2% (34.3, 46.3)	35.1% (29.2, 41.5)	37.5% (34.2, 40.9)
	14 years	30.1% (23.4, 37.7)	23.8% (17.6, 31.4)	26.9% (22.6, 31.7)	25.3% (19.8, 31.6)	28.1% (22.6, 34.3)	26.7% (23.0, 30.9)
Highest parents/caregivers’ educational attainment	Less than primary sch.	1.8% (1.0, 3.4)	2.6% (1.9, 7.1)	2.2% (1.0, 4.7)	6.2% (3.2, 11.6)	11.3% (7.6, 16.4)	8.8% (5.9, 13.1)
	Completed primary sch.	4.7% (2.1, 10.0)	6.1% (2.6, 13.7)	5.4% (2.5, 11.3)	7.4% (4.9, 11.1)	8.7% (5.8, 12.8)	8.1% (6.1, 10.6)
	Completed middle sch.	25.5% (17.6, 35.6)	26.4% (18.4, 36.3)	26.0% (18.6, 34.9)	26.3% (17.8, 37.0)	25.2% (19.8, 31.4)	25.7% (19.6, 33.0)
	Completed secondary school	45.4% (35.7, 55.3)	44.2% (34.8, 53.9)	44.8% (36.9, 52.9)	39.3% (31.0, 48.3)	30.7% (23.4, 39.2)	34.9% (29.2, 41.0)
	College graduate or more	22.6% (12.7, 37.0)	20.8% (12.5,32.4)	21.7% (13.5, 33.0)	20.8% (15.2, 27.7)	24.1% (15.0, 36.5)	22.5% (16.2, 30.4)
Religious affiliation	Hindu	71.5% (59.7, 80.9)	74.6% (60.9, 84.8)	73.1% (61.3, 82.3)	80.7% (61.0, 91.8)	80.4% (64.5, 90.2)	80.5% (63.5, 90.8)
	Muslim	17.8% (8.9, 32.4)	16.8% (7.5, 33.4)	17.3% (8.6, 31.8)	18.3% (7.5, 38.3)	19.3% (9.5, 35.2)	18.8% (8.7, 36.1)
	Other (Zoroastrian, etc.)	10.7% (5.8, 19.0)	8.6% (5.0, 14.5)	9.6% (5.8, 15.6)	1.0% (0.3, 0.0)	0.3% (0.1, 1.7)	0.6% (0.2, 1.8)
Adolescent choice of language of interview	Hindi	63.2% (51.0, 74.0)	52.9% (43.7, 61.9)	58.0% (48.9, 66.6)	8.5% (4.7, 15.0)	7.3% (4.1, 12.7)	7.9% (5.1, 12.1)
	English	8.2% (3.7, 17.4)	7.6% (3.2, 17.1)	7.9% (3.9, 15.3)	10.4% (5.4, 18.9)	7.5% (3.1, 16.9)	8.8% (4.7, 16.0)
	Regional language	28.6% (19.5, 39.8)	39.5% (29.5, 50.5)	34.0% (26.0, 43.0)	81.1% (72.2, 87.7)	85.2% (75.4, 91.6)	83.3% (75.3, 89.0)
Type of school attended	Private	82.6% (72.8, 89.4)	72.5% (60.6, 81.9)	77.6% (67.0, 85.5)	37.4% (28.6, 47.2)	30.0% (22.1, 39.3)	33.5% (26.1, 41.9)
	Government	12.4% (7.4, 19.9)	20.0% (13.2, 29.0)	16.2% (10.6, 24.0)	54.2% (46.9, 61.3)	57.7% (50.8,64.3)	56.0% (50.6, 61.3)
	Government-aided	5.0% (2.7, 8.9)	7.5% (4.3, 12.8)	6.3% (3.7, 10.4)	8.4% (3.5, 18.8)	12.3% (7.2, 20.3)	10.4% (6.2, 17.2)

*Note. Sample sizes slightly reduced for individual measures because of missing data

^a^ Wave 1 sample size excludes 10 Mumbai adolescent participants who reported past tobacco use

^b^ Wave 1 sample size excludes 8 Kolkata adolescent participants who reported past tobacco use

Table 2 describes the prevalence of susceptibility to smoking or smokeless tobacco use for males and females, respectively, among those who had never experienced prior tobacco use. The susceptibility prevalence by age, by sex-at-birth and within city did not vary significantly except for lower susceptibility to smoking cigarettes for Kolkata females (chi square(2) = 30.5, *p* =.0016). Susceptibility to future smoking and susceptibility to future smokeless tobacco use were highly correlated (phi = 0.75; *p* <.0001). For the main analyses examining the association of prosocial factors with adolescent susceptibility to tobacco use, therefore, only susceptibility to total tobacco use (combining susceptibility to smoking and smokeless tobacco use) is reported. The supplemental tables present results specific to susceptibility to smoking or to smokeless tobacco use (see Additional File 2).

**Table 2:** Non-tobacco-using males’ and females’ susceptibility to future tobacco use (weighted), by wave and by city (%, 95% confidence interval)

Susceptibility measures	Wave	Mumbai (weighted)			Kolkata (weighted)		
		Males *n*^d^ = 475	Females *n*^d^ = 459	Total *N* = 934	Males *n*^d^ = 500	Females *n*^d^ = 522	Total *N* = 1022
		weighted % (95% CI)	weighted % (95% CI)	weighted % (95% CI)	weighted % (95% CI)	weighted % (95% CI)	weighted % (95% CI)
Susceptible to smoking^a^	Wave 1	12.2% (7.2, 19.9)	3.2% (1.4, 7.1)	7.7% (4.6, 12.5)	8.0% (4.5, 13.7)	6.3% (2.9, 12.9)	7.1% (4.5, 11.0)
	Wave 2	17.2% (10.9, 26.1)	21.6% (14.4, 31.2)	19.3% (13.0, 27.7)	12.8% (9.1, 17.7)	11.8% (7.4, 18.1)	12.3% (9.4, 15.9)
Susceptible to chewing or applying smokeless tobacco^b^	Wave 1	10.0% (6.0, 16.2)	4.2% (2.1, 8.0)	7.1% (4.3, 11.4)	6.3% (3.9, 9.8)	3.4% (1.6, 7.2)	4.8% (3.0, 7.6)
	Wave 2	13.6% (7.8, 22.5)	14.6% (8.5, 23.8)	14.0% (8.4, 22.4)	12.0% (7.8, 18.0)	6.2% (3.1, 12.1)	9.1% (6.4, 12.6)
Susceptible to using either smoking or smokeless tobacco	Wave 1	12.6% (7.5, 22.3)	4.5% (2.4, 8.5)	8.6% (5.2, 13.8)	9.4% (5.8, 15.1)	7.1% (3.6, 13.7)	8.2% (5.4, 12.3)
	Wave 2	19.1% (12.1, 28.7)	21.9% (14.6, 31.6)	20.4% (13.8 29.1)	16.0% (11.8, 21.4)	12.3 (7.8, 19.1)	14.2% (11.1, 17.8)

^a^Susceptibility to smoking as indicated by any answer other than “Definitely not” to the question “At any time during the next 12 months, do you think you will smoke a cigarette or bidi?;” limited to participants who were never smokers at wave 1

^b^Susceptibility to smokeless tobacco use as indicated by any answer other than “Definitely not” to the question, “At any time during the next 12 months, do you think you will chew or apply tobacco in any form?“; limited to participants who had never used chewing tobacco at wave 1 assessment

Susceptibility to either smoking or smokeless tobacco use combined, derived from susceptibility to smoking + susceptibility to smokeless tobacco use; limited to wave 1 never-users of any tobacco product

^d^Sample size at wave 1

Table 3 describes the prosocial factors, including the participants’ W1 school connectedness, the frequency with which W1 tobacco-naive participants prayed at home, how important they rated daily prayer, and how often they attended a place of worship.

**Table 3:** City-specific W1 and W2 prevalence of respondent attachment to their school and three measures of respondent level of religiosity

Prosocial measures	Wave	Answer options	Mumbai (weighted)			Kolkata (weighted)		
			Males	Females	Total	Males	Females	Total
			n^a^ = 480	n^a^= 454	*N* = 934	n^b^ = 502	n^b^= 528	*N* = 1,030
			weighted % (95% CI)	weighted % (95% CI)	weighted % (95% CI)	weighted % (95% CI)	weighted % (95% CI)	weighted % (95% CI)
You feel part of your school	W1	Disagree	1.0% (0.4, 2.6)	0.1% (0.0, 0.6)	0.5% (0.2, 1.4)	1.2% (0.4, 3.1)	0.8% (0.3, 2.1)	0.9% (0.5, 1.9)
		Neither agree nor disagree	2.6% (1.3, 5.0)	2.7% (1.0, 7.0)	2.6% (1.4, 5.0)	6.2% (3.3, 11.2)	5.0% (2.5, 10.0)	5.6% (3.4, 9.1)
		Agree	96.4% (93.4, 98.0)	97.2% (92.9, 98.9)	96.8% (94.2, 98.3)	92.7% (87.4, 95.8)	94.2% (88.8, 97.1)	93.5% (89.8, 95.9)
	W2	Disagree	2.1% (0.6, 7.1)	0.7% (0.1, 3.4)	1.4% (0.4, 5.2)	1.3% (0.4, 4.4)	0.4% (0.1, 1.6)	0.8% (0.3, 2.8)
		Neither agree nor disagree	2.4% (1.1, 5.3)	1.0% (0.4, 2.7)	1.8% (0.9, 3.3)	12.2% (9.5, 15.6)	9.7% (6.9, 13.6)	11.0% (8.6, 13.9)
		Agree	95.5% (90.1, 98.0)	98.3% (96.5, 99.2)	96.8% (93.7, 98.4)	86.5% (82.9, 89.4)	89.9% (86.1, 92.7)	88.2% (85.3, 90.6)
You are happy to be at school	W1	Disagree	0.1% (0.0, 1.0)	0.2% (0.1, 0.6)	0.2% (0.1, 0.5)	1.6% (0.8, 3.1)	1.2% (0.5, 2.8)	1.4% (0.7, 2.6)
		Neither agree nor disagree	3.6% (1.5, 8.2)	0.7% (0.2, 2.5)	2.1% (1.0, 4.3)	7.7% (5.2, 11.1)	3.0% (1.5, 5.6)	5.2% (3.7, 7.2)
		Agree	96.3% (91.7, 98.4)	99.1% (97.6, 99.7)	97.7% (95.6, 98.8)	90.7% (87.4, 93.3)	95.9% (93.2, 97.6)	93.4% (91.6, 94.9)
	W2	Disagree	1.7% (0.9, 3.2)	0.3% (0.0, 2.5)	1.0% (0.6, 1.8)	1.2% (0.5, 3.3)	0.5% (0.1, 3.8)	0.9% {0.3, 2.2)
		Neither agree nor disagree	3.6% (2.2, 5.8)	2.7% (1.4, 5.2)	3.2% (2.2, 4.5)	4.6% (2.3, 9.0)	5.8% (3.7, 8.9)	5.2% (3.4, 7.9)
		Agree	94.7% (92.9, 96.1)	97.0% (94.1, 98.5)	95.8% (94.4, 96.8)	94.1% (89.8, 96.7)	93.7% (90.1, 96.1)	93.9% (91.2, 95.9)
You feel safe in your school	W1	Disagree	0.1% (0.0, 0.9)	0.5% (0.1, 2.5)	0.3% (0.1, 1.1)	1.2% (0.4, 3.2)	0.9% (0.3, 2.2)	1.0% (0.5, 2.1)
		Neither agree nor disagree	3.3% (1.4, 7.7)	0.5% (0.1, 2.6)	1.9% (0.8, 4.2)	4.7% (2.5, 8.8)	0.4% (0.1, 1.4)	2.5% (1.4, 4.4)
		Agree	96.6% (92.2, 98.5)	99.0% (97.2, 99.7)	97.8% (95.7, 98.9)	94.1% (90.1, 96.5)	98.7% (97.2, 99.4)	96.5% (94.8, 97.7)
	W2	Disagree	1.9% (0.8, 4.5)	1.5% (0.5, 4.0)	1.7% (0.8, 3.4)	1.7% (0.6, 5.3)	0.5% (0.1, 3.8)	1.1% (0.4, 3.0)
		Neither agree nor disagree	3.4% (2.0, 5.8)	1.4% (0.4, 4.1)	2.4% (1.4, 4.3)	7.3% (3.9, 13.3)	6.3% (3.9, 10.0)	6.8% (4.5, 10.1)
		Agree	94.7% (91.3, 97.2)	96.8% (92.5, 99.0)	95.8% (92.8, 97.6)	90.9% (85.1, 94.6)	93.2% (89.7, 95.6)	92.0% (88.6, 94.5)
Frequency of prayer	W1	1–3 times/month or less	16.4% (12.6, 21.2)	16.9% (10.4, 26.2)	16.7% (12.1, 22.6)	52.9% (42.7, 62.9)	36.8% (28.8, 45.6)	44.5% (36.7, 52.5)
		1–3 times a week	33.0% (20.6, 48.4)	28.5% (16.0, 45.5)	30.8% (18.8, 46.1)	25.7% (20.5, 31.6)	33.0% (25.7, 41.3)	29.6% (24.2, 35.6)
		Nearly every day	50.5% (36.7, 64.3)	54.5% (43.6, 65.1)	52.5% (41.0, 63.8)	21.4% (14.0, 31.2)	30.1% (22.6, 38.9)	26.0% (20.2, 32.8)
Importance of prayer	W1	Not at all important	3.0% (1.5, 6.1)	1.6% (0.7, 3.4)	2.3% (1.3, 4.1)	0% (0.0, 0.03)	0% (0.0, 0.0)	0% (0.0, 0.01)
		Somewhat important	31.2% (22.3, 41.6)	17.9% (11.7, 26.4)	24.5% (18.0, 32.4)	49.2% (38.6, 59.8)	33.2% (24.6, 43.0)	40.8% (32.5, 49.6)
		Very important	65.8% (56.2, 74.3)	80.6% (72.2, 86.9)	73.2% (65.6, 79.6)	50.8% (40.2, 61.4)	66.8% (57.0, 75.4)	59.2% (50.4, 67.5)
Frequency of attendance at place of worship	W1	Never	10.7% (5.6, 19.4)	7.3% (3.6, 14.3)	9.0% (4.7, 16.4)	12.4% (4.3, 30.7)	12.5% (5.2, 27.3)	12.5% (4.9, 28.3)
		1–3 times a year	13.1% (8.6, 19.8)	16.0% (8.7, 27.7)	14.6% (9.1, 22.6)	33.6% (27.3, 40.6)	31.7% (22.6, 42.4)	32.6% (26.0, 39.9)
		1–3 times a month	36.0% (25.0, 48.8)	32.2% (22.8, 43.4)	34.1% (24.6, 45.2)	39.3% (31.2, 48.1)	42.8% (30.7, 55.8)	41.2% (32.4, 50.5)
		1–3 times a week	25.4% (17.6, 35.2)	28.8% (20.8, 38.4)	27.1% (20.4, 35.0)	9.7% (6.6, 14.1)	7.7% (4.3, 13.3)	8.6% (6.4, 11.6
		Nearly every day	14.7% (8.7, 23.8)	15.6% (9.4, 24.7)	15.1% (9.6, 23.1)	5.0% (2.5, 9.6)	5.3% (2.8, 10.0)	5.2% (2.9, 9.2)

^a^ Wave 1 sample size excludes 10 Mumbai adolescent participants who reported past tobacco use

^b^ Wave 1 sample size excludes 8 Kolkata adolescent participants who reported past tobacco use

### School connectedness

 The magnitude of the correlations between the three school connectedness measures (Cronbach alpha ranged from 0.45 to 0.69 across the two cities and over the two waves) were high enough to warrant these three measures being summed. Only analyses of the summative score are reported in regression analyses. Because the summative score was highly skewed, with few respondents disagreeing with the statement that they felt well-connected to their school, the summative score was dichotomized, with any disagreement coded as zero and consistent agreement coded as one. Most respondents (> 79%) in both cities in both waves reported feeling well-connected to their school, but Mumbai respondents in W2 were more likely to agree that they felt well-connected to their school (90.4%; 95%CI: 86.2, 93.4) than Kolkata respondents (79.4%; 95%CI:75.5, 82.8; *p* =.001). To ensure a fair contrast between the two cities, propensity scores were applied, with matching variables including adolescent sex-at-birth, preferred language, age, religious affiliation, and parent/guardian highest educational attainment. Treating city as the treatment effect, Kolkata’s mean percent agreement that respondents felt well-connected to their school was significantly less than Mumbai’s (coefficient = −0.10; 95%CI: −0.15, −0.05; *p* <.001).

### Three measures of religiosity

 For all three measures of religiosity, residents of Mumbai scored higher on religiosity than Kolkata residents. Residents of Mumbai prayed more often than residents of Kolkata (F(2.66, 119.61) = 11.0, *p* <.0001). More than twice the percentage of Mumbai adolescents (52.5%; CI: 41.1, 63.6) as Kolkata adolescents (25.9%; CI: 20.1, 32.7) reported praying nearly every day. Nearly three quarters (73.0%; CI: 65.4, 79.4) of Mumbai adolescents said that prayer was “very important” in contrast to only 59.4% (CI: 50.4, 67.7) of Kolkata adolescents. Finally, 42.2% (CI: 33.4, 51.6) of Mumbai adolescents attended a place of worship at least weekly whereas the corresponding figure for Kolkata adolescents was only 13.9% (CI: 10.0, 18.9). To ensure a fair contrast between the two cities, propensity matching scores were applied, with matching variables including adolescent sex-at-birth, preferred language, age, religious affiliation, and parent/guardian highest educational attainment. Treating city as the treatment effect, Kolkata’s prevalence of prayer frequency was significantly less than Mumbai’s (coefficient = −0.34; CI: −0.46, −0.21; *p* <.001), as was Kolkata’s frequency of attendance at a mosque or temple (coefficient = −0.79; 95% CI: −0.94, −0.64; *p* <.001). The prevalence of prayer importance, however, did not differ between the two cities after propensity matching scores were applied.

#### W1 school connectedness as a predictor of W1 tobacco use susceptibility

For Mumbai, there was a significant main effect of school connectedness (aOR = 0.29, CI: 11.1, 78.2; *P* =.015) with 15.3% (CI: 5.5, 25.1) of those who were ambivalent about feeling connected to their school reporting tobacco use susceptibility compared to 7.7% (CI: 3.8, 11.6) of those who reported full agreement that they felt well-connected to their school. Sex-at-birth was a significant covariate (aOR = 0.02; CI: 0.00, 22.8; *p* =.003), with only 4.3% (CI: 1.5, 7.0) of females reporting susceptibility to future tobacco use compared to 12.7% (CI: 6.7, 18.7) of males reporting susceptibility to future tobacco use. For Kolkata, school connectedness was associated with neither W1 tobacco use susceptibility nor sex-at-birth.

#### W1 frequency of prayer as a predictor of W1 tobacco use susceptibility

Type of school (private, government, government-aided schools) was unrelated to adolescents’ frequency of prayer in Mumbai but was significantly related to adolescents’ frequency of prayer in Kolkata (F(2, 21) = 10.05; *p* =.009). Frequency of prayer was lowest in Kolkata’s private schools (marginal mean (mm) = 3.35; 95% CI: 3.05, 3.66); intermediate in Kolkata’s secular government schools (mm = 3.53; 95% CI: 3.29, 3.77) and highest in Kolkata’s government-aided schools (mm = 3.97; 95% CI: 3.68, 4.26). School type was therefore included as a covariate in all regressions involving frequency of prayer as a regressor for both cities.

There was a quadratic effect of prayer frequency (F(1, 45) = 4.70; *p* =.0355) on W1 susceptibility to tobacco use in Mumbai males with 27.7% (95% CI: 8.9, 46.6) of males identified as susceptible when they reported praying 1–3 times per month or less frequently, 6.8% (0.4, 13.2) as susceptible when praying 1–3 times per week and 11.5% (4.5, 18.6) as susceptible when praying nearly every day. Mumbai females’ susceptibility to tobacco use was unrelated to their W1 frequency of prayer. Sex-at-birth was a significant correlate of susceptibility to tobacco use in Mumbai (aOR = 0.16; 95% CI: 0.04, 0.73; *p* =.019)), with males being overall more susceptible (15.0%; 95% CI: 7.4, 22.5) than females (4.6%; 95% CI: 1.5, 7.7). In Kolkata, prayer frequency was unrelated to adolescents’ susceptibility to using tobacco.

#### W1 importance of prayer and W1 tobacco use susceptibility

In Mumbai, there was an inverse association between adolescents’ importance of prayer and their susceptibility to initiating use of any type of tobacco (F(2,44) = 17.79; *p* <.0001). The percentages reporting susceptibility to tobacco by level of prayer importance were: 33.2% (Not at all important; CI: 13.4, 53.1), 10.8% (Somewhat important; CI: 3.1, 18.5) and 6.5% (Very important; CI: 3.0, 9.9). Males and females did not differ in this association. In Kolkata, there was no statistically significant association between adolescents’ reported W1 importance of prayer and their susceptibility to using tobacco.

#### W1 frequency of attendance at a place of worship and W1 tobacco use susceptibility

In Mumbai, there was a significant interaction between sex-at-birth and frequency of attendance at a place of worship (F(4, 24) = 3.94; *P* =.0084). Female adolescents reported non-significantly different levels of tobacco use susceptibility to tobacco use compared to males when they reported rarely attending a house of worship (2.5% when almost never attending; CI: −0.01,5.9) versus 13.6% for male adolescents (CI: −1.9%, 29.0; 6.3% when attending only 1–3 times/year; CI: −2.2%, 14.7) versus 16.2% for male adolescents (CI: 2.2, 30.2) but significantly lower levels than male adolescents when they reported more frequent attendance at a house of worship (3.5% when attending 1–3 times/month; CI: −0.00, 7.2) versus 8.1% for male adolescents (CI: 2.0, 14.2); 1.6% when attending 1–3 times/week; CI: −0.01, 3.8) versus 14.0% for male adolescents (CI: 4.3, 23.7); 2.8% when attending nearly every day: CI: −0.02, 7.3) versus 14.8% for male adolescents (CI: −0.03, 33.0). In Kolkata there was no statistically significant association between W1 frequency of attendance at a place of worship and W1 susceptibility to using any form of tobacco.

#### Change from W1 to W2 in tobacco use susceptibility as reflected by reduction in proportion of respondents who consistently said “Definitely not”

 Adolescent never-tobacco-users’ one-year change in susceptibility to any tobacco use (smoking or smokeless tobacco use combined) increased significantly (F(1, 45) = 7.34, *P* =.0095) in Mumbai from 9.8% (CI: 6.3, 14.3) at W1 to 20.4% (CI: 14.2, 29.1) at W2 but failed to change significantly from 10.3% (CI: 7.9, 13.3) at W1 to 14.2% (CI: 11.1, 18.0) at W2 in Kolkata.

#### W1 school connectedness as predictor of change in tobacco use susceptibility from W1 to W2 in Mumbai

In Mumbai, there was a main effect of school connectedness on W1 to W2 change in adolescent susceptibility to future tobacco use (F(1, 45) = 5.50; *P* =.0235) with respondents who felt well-connected to their school reporting significantly less increased tobacco use susceptibility to future tobacco use than respondents who felt ambivalent about their school. No other effects were observed. In Kolkata, there was a significant interaction effect of W1 school connectedness with sex-at-birth on W1 to W2 change in tobacco use susceptibility (F(1, 45) = 6.54, *P* =.006). W1 to W2 change in tobacco use susceptibility was nearly identical for males and females who reported feeling well-connected to their school whereas males who expressed ambivalence about feeling well-connected to their school registered a significantly increased susceptibility to tobacco use compared to females who expressed ambivalence about their school (F(1, 45) = 17.71; *P* =.0001).

#### Effect of W1-W2 change in school connectedness on W1-W2 change in adolescent susceptibility to tobacco use

In Mumbai, W1 to W2 change in school connectedness was associated with W1 to W2 change in susceptibility to tobacco use (F(1, 45) = 6.21, *P* =.0164) for both males (F(1, 45) = 6.21, *P* =.0164) and females (F(1, 45) = 11.75, *P* =.0013). For the 14.7% of adolescents who expressed any ambivalence at any time about feeling well-connected to their school, the probability of increased susceptibility to tobacco use was 45.5% (CI: 31.8, 59.3), more than double the 22.4% (CI: 14.2, 30.5) increased susceptibility to tobacco use reported by the 85.3% of adolescents who consistently reported feeling well-connected to their school. In Kolkata, there was no significant association between change in school connectedness and change in tobacco use susceptibility.

#### W1 religiosity as predictor of change in tobacco use susceptibility from W1 to W2 

Mumbai W1 frequency of prayer was not associated with W1 to W2 change in susceptibility to future tobacco use. Kolkata adolescents’ change in tobacco use susceptibility was also unrelated to the change in their W1-W2 frequency of prayer.

Mumbai male adolescent W1 to W2 change in tobacco use susceptibility increased linearly with declining W1 importance of prayer (F(1, 45) = 8.36; *P* =.0059). The increases in tobacco use susceptibility were 51.4% (95% CI: 28.0, 74.8) for those reporting that prayer was not at all important, 31.2% (95% CI: 21.9, 40.5) for those reporting that prayer was somewhat important and 25.6% (95% CI: 14.6, 36.5) for those reporting that prayer was very important. Prayer importance was unrelated to the W1 to W2 change in tobacco use susceptibility for Mumbai females. While there was no main effect of prayer importance on W1 to W2 change in tobacco use susceptibility for Kolkata adolescents, there was a simple main effect for females (F(1, 45) = 6.82; *P* =.0122). The W1 to W2 increase in female risk of tobacco use susceptibility was greater for those whose religiosity was not very important (25.2%; CI: 15.0, 35.4) compared to those whose religiosity was very important (14.7%; CI: 9.8, 19.6).

In Mumbai, there was a main effect for frequency of attendance at a place of worship on W1 to W2 change in tobacco use susceptibility to any type of tobacco use (F(4,42) = 2.96, *P* =.0304) but within the sex-at-birth categories, only the males exhibited a statistically significant downward linear trend (F(1, 45) = 5.97; *P* =.0185). Male adolescents’ W1 frequency of attendance at a place of worship was associated with a reduction in probability of increasing their risk of tobacco use susceptibility from W1 to W2 when comparing those who almost never attended a place of worship (31.4%; CI: 21.7, 41.2) with those who attended a place of worship nearly every day (15.9%; CI: 0.00, 31.5).

*In Kolkata*,* the W1 to* W2 change in susceptibility to tobacco use was not associated with frequency of attendance at a place of worship.

## Discussion

As predicted by Problem Behavior Theory [37] and Resiliency Theory [39], prosocial factors relating to school connectedness and religiosity were generally but not always associated with reduced susceptibility to tobacco use in Mumbai. Interactions of these prosocial factors with sex-at-birth were occasionally significant, with females usually at lower risk at W1 and at lower risk of increased susceptibility from W1 to W2 in Kolkata than males. While trends were generally in the predicted direction in Kolkata, they were statistically significant only for female importance of prayer. Religiosity appeared to be a statistically significant but often relatively small influence on both W1 cross-sectional and one-year change in susceptibility to tobacco use in Mumbai and even smaller influence in Kolkata.

The lower risk of tobacco use susceptibility observed in females compared to males in both cities is consistent with 2019 youth (13–15 year olds) tobacco use epidemiology specific to Maharashtra and West Bengal, the two states in which Mumbai and Kolkata are located, respectively [5, 6]. Perhaps because female risk of tobacco use susceptibility was consistently lower and less variable than male risk, significant interaction effects with religiosity or school connectedness tended to involve males, not females.

The finding that Kolkata’s adolescents reported significantly lower religiosity than Mumbai’s adolescents could be explained by Kolkata’s historical support for secularism [31], rationalism, humanism and questioning of traditional practices during the 19th and early 20th century “Bengal Renaissance” [110, 111]. The higher apparent religiosity observed in Mumbai dates back to the late 19th century when freedom fighters popularized the public festival of Ganesh Chaturthi as a way to foster a sense of unity and nationalism among Indians during British rule [112]. The higher reported school connectedness reported by Mumbai respondents could reflect the higher proportion of Mumbai respondents (58%) who preferred to speak Hindi compared to the Kolkata respondents (16%). Regional language speakers tend to have less access to higher education-related opportunities than national language speakers [113, 114]. These disparate findings suggest that cultural context moderates the association between religiosity and adolescent tobacco use susceptibility.

Our results extend findings from U.S. and European studies to the India context by showing a generally inverse association between adolescent tobacco use susceptibility and frequency of attendance at a place of worship for Mumbai males [31, 102, 103]. In India, where religion plays a pervasive role in society [36], it is not surprising that adolescent religiosity could have a protective effect against tobacco use initiation. However, the scientific literature has documented that the relationship between religiosity and health behaviors is complex and influenced by additional cultural, social, and individual factors [31, 36]. While adolescent religiosity may protect adolescents from tobacco use initiation, other factors such as family socioeconomic status [115], peer influence [116], and access to tobacco products [117] may also play significant roles in shaping adolescent tobacco use susceptibility but were not tested in this paper. When included with these more traditional risk factors to future tobacco use, adolescents’ religiosity, involvement in faith-based activities, and involvement in school-based activities have been shown to improve identification of adolescents who initiate cigarette smoking [118].

Future waves of data from the participants in the present study may provide more insight into how sex-at-birth and city-specific local cultural attitudes toward prosocial factors may modify the impact of prosocial factors such as school connectedness and religiosity on susceptibility to tobacco use in Mumbai and Kolkata adolescents.

### Study strengths and limitations

Strengths of this study include population-based sampling, examining the religiosity-tobacco use question among mostly non-Christian respondents [59], longitudinal tests of hypotheses [119], use of multiple indicators of religiosity, household interview data, the use of computer-aided personal interviewing for the collection of sensitive data, a relatively high (88.6%) study retention rate over two years and conducting the survey in multiple languages to ensure broad representation of the population. Other strengths include testing interactions with sex-at-birth and stratifying analyses by city. Weaknesses of the study include the lack of biological validation of respondent tobacco use status and the limited number of theoretically important psychosocial predictors of adolescent tobacco use initiation. Fortunately, past research has shown that adolescent self-reports of tobacco use status tend to be accurate [120].

## Conclusions

If the foregoing results are confirmed in subsequent research, there may be benefit in having public health professionals partner with school leaders, community leaders and leaders in the faith community to deliver anti-tobacco health education [76, 121]. Partnering with schools to ensure that all students feel well-connected to their schools could yield multiple societal benefits in addition to tobacco use prevention [100]. Prayer may be an underappreciated, accessible tool for adolescents to use in lieu of nicotine delivery devices for coping with typical developmental stressors [122] characteristic of adolescence [51].

## Supplementary Information

Supplementary Material 1.

Supplementary Material 2.

Supplementary Material 3.

## Data Availability

The authors will share as much of the deidentified data as the government of India will permit upon researcher request.
