# Supplementary material for: Religiosity, school connectedness, and tobacco use susceptibility: a longitudinal study of adolescents in Mumbai and Kolkata, India
Source: BMC Public Health. 2025 Nov 7;25:3857. doi: 10.1186/s12889-025-25035-7 (PMC12595895; doi:10.1186/s12889-025-25035-7)
Supplement: Supplementary file 1 — Supplementary Material 1. [file 12889_2025_25035_MOESM1_ESM.pdf]

Supplemental file #1 – Selected items from the English version of the W1 adolescent questionnaire.

Selected questions from the English version of the W1 adolescent questionnaire used in this study

PLEASE DO NOT READ OUT THE RESPONSE OPTIONS TO THE RESPONDENT UNLESS IT SAYS "READ OUT RESPONSE OPTIONS". PROVIDE RESPONSE CARDS TO PARTICIPANTS WHEREVER INDICATED AND ASK THEM TO REPOND TO THE QUESTIONS ACCORDINGLY.

Thank you for agreeing to be a part of our study. I will now ask you questions for about 60 minutes.

- **Please listen to each question carefully before answering it.**
- **Remember this is not a test and there are no right or wrong answers. We want you to answer based on what you really know and do.**
- **Answer the questions truthfully and as best as you can.**
- **Response options will be provided wherever needed to help you answer the question. Listen to or read all the response options carefully before answering. Don't choose a response just because you think that's what someone wants you to say.**
- **It would be best if you answered all questions, but you can skip if you do not want to answer a question.**
- **The interview is private and confidential. We will not share your answers with anyone.**

Section A: DEMOGRAPHICS

First, I will ask you a few questions about your background.

A1. Gender? (OBSERVATION ONLY)
Male (1)
Female (2)
A2. What is your date of birth? (DD-MM-YYYY)

- ❍ Day _ _
- ❍ Month _ _
- ❍ Year _ _

A3. What grade are you in?

________ (GRADE 1 -12)
INPUT 0 IF RESPONDENT DOES NOT ATTEND SCHOOL

CONDITION: IF NOT ATTENDING SCHOOL (0) - SKIP TO A5

A4. What percentage did you get last school year?

________ (PERCENTAGE 0-100)

A5. What is the highest class or standard you finished?

- ❍ Never attended school (1)
- ❍ Specify Class/Standard _________ (2)

A6. Did you work outside the home for money, in the last 12 months?

- ❍ Yes (1)
- ❍ No (2)

Section B: TOBACCO-RELATED FACTORS

Now I am going to ask a few questions about tobacco use. Tobacco can be smoked as cigarette, bidi, cigar, chutta, dhumti, or it can be smoked in hukka, chilum, pipe etc. It can be chewed as gutka, pan masala, betel-quid, khaini, mawa, zarda, or applied to the gums as mishri, gul, bajjar, snuff, tobacco toothpaste, tobacco tooth powder, and other products.

B1. Have you ever tried or experimented with any form of tobacco, even once or twice? (READ OUT RESPONSE OPTIONS)

- ❍ Never (0)
- ❍ Once or twice (1)
- ❍ Three or more times (2)

CONDITION: IF NEVER (0) IS SELECTED. SKIP TO: B7

B2. How old were you when you first tried tobacco in any form?

_______________years old

B3. When you first tried or experimented with tobacco, which form of tobacco did you use?

- ❍ Cigarrete
- ❍ Bidi
- ❍ Chutta
- ❍ Hookah
- ❍ Gutkha
- ❍ Pan masala
- ❍ Zarda
- ❍ Mishri
- ❍ Snuff
- ❍ Tobacco toothpaste or tooth powder
- ❍ Other (SPECIFY): ________

B4. During the past 30 days, did you <u>smoke any</u> tobacco product?

- ❍ Yes (1)
- ❍ No (2)

B4.1 During the past 30 days, which of the following tobacco products did you smoke? (READ OUT PRODUCTS)

CONDITION: IF B4=1

	Yes	No
a) Cigarettes?	❍	❍
b) Bidis?	❍	❍
c) Cigars?	❍	❍
d) Chutta or Dhumti?	❍	❍
e) Hookah or water pipe?	❍	❍
f) Pipe?	❍	❍
g) Other forms?	❍	❍
If yes, SPECIFY: _________		

B4.2 During the past 30 days, on how many days did you smoke tobacco in any form?

_____________days

CONDITION: IF B4=1

B5. During the past 30 days, did you chew any tobacco product?

- ❍ Yes (1)
- ❍ No (2)

B5.1 During the past 30 days, <u>which of the following</u> tobacco products did you <u>chew?</u> (READ OUT PRODUCTS)

CONDITION: IF B5=1

	Yes	No
a) Tobacco by itself?	❍	❍
b) Tobacco lime mixture without supari?	❍	❍
c) Pan masala with tobacco?	❍	❍
d) Pan with tobacco or tobacco-lime supari mixtures?	❍	❍
e) Gutkha?	❍	❍
f) Other forms? If yes, SPECIFY: _________	❍	❍

B5.2 During the past 30 days, on how many days did you <u>chew</u> tobacco in any form?

____

CONDITION: IF B5=1

B6. During the past 30 days, did you <u>apply any</u> tobacco products to your gums?

- ❍ Yes (1)
- ❍ No (2)

B6.1 During the past 30 days, <u>which of the following</u> tobacco products did you <u>apply?</u> (READ OUT PRODUCTS)

CONDITION: IF B6=1

	Yes	No
a) Mishri?	❍	❍
b) Snuff, Bajjar or Tapkir?	❍	❍
c) Tobacco toothpaste (e.g. Ipco or Dentobac)	❍	❍
d) Tobacco toothpowder (e.g. Lal dantamanjan)	❍	❍
e) Other forms?<br>If yes, SPECIFY: ________	❍	❍

B6.2. During the past 30 days, on how many days did you <u>apply</u> tobacco to your gums in any form?

____

CONDITION: IF B6=1

The following questions are about e-cigarettes.

B7. Have you heard of e-cigarettes?

❍ Yes (1)
❍ No (2)

B7.1. Have you ever tried or experimented with e-cigarettes even once or twice?

- ❍ Yes (1)
- ❍ No (2)

CONDITION: IF B7=1

B8. You mentioned you used more than one tobacco product during the last 30 days? Which of these products did you use most often? (READ OUT OPTIONS)

- ❍ Cigarettes
- ❍ Bidis
- ❍ Cigars
- ❍ Chutta or Dhumti
- ❍ Hookah or water pipe
- ❍ Pipe
- ❍ Chew tobacco by itself
- ❍ Tobacco lime mixture without supari
- ❍ Pan masala with tobacco
- ❍ Pan with tobacco or tobacco-lime supari mixtures
- ❍ Gutkha
- ❍ Mishri
- ❍ Snuff, Bajjar or Tapkir
- ❍ Tobacco toothpaste (e.g. Ipco or Dentobac)
- ❍ Tobacco toothpowder (e.g. Lal dantamanjan)
- ❍ Other forms, SPECIFY _________

B9. During the past 30 days, how did you usually get your tobacco in any form? (SELECT ALL THAT APPLY) (READ OUT RESPONSE OPTIONS)

- ❑ I bought them in a store or shop or street vendor (1)
- ❑ I got tobacco from someone else (2)
- ❑ It was available at home (3)
- ❑ I got them in some other way. SPECIFY ____ (4)

CONDITION: IF USED TOBACCO IN THE LAST 30 days

B10. During the past 30 days, did anyone ever refuse to sell you cigarettes or bidis because of your age?

- ❍ Yes (1)
- ❍ No (2)
- ❍ I did not try to buy cigarettes or bidis during the past 30 days (3)

CONDITION: IF USED TOBACCO IN THE LAST 30 days

B11. During the past 30 days, did anyone ever refuse to sell you smokeless tobacco products because of your age?

- ❍ Yes (1)
- ❍ No (2)
- ❍ I did not try to buy any smokeless tobacco products during the past 30 days (3)

CONDITION: IF USED TOBACCO IN THE LAST 30 days

B12. Have you seen kids your age buying any tobacco products such as cigarettes, bidis, mishri, khaini etc.?

- ❍ Yes (1)
- ❍ No (2)

B13. Have you seen kids your age buying gutka?

- ❍ Yes (1)
- ❍ No (2)

The next questions ask about stopping tobacco use.

B14. In the past 12 months, have you ever tried to stop using tobacco?

❍ Yes (1)
❍ No (2)

CONDITION: IF USED TOBACCO IN THE LAST 30 days

B15. Do you think you would be able to stop using tobacco if you wanted to? (READ OUT RESPONSE OPTIONS, IF NECESSARY))

❍ Yes, I can stop if I want (1)
❍ No, I don't think I will be able to stop (2)
❍ I have already stopped using tobacco (3)

CONDITION: IF USED TOBACCO IN THE LAST 30 days

B16. Have you ever received help or advice to help you stop using tobacco? (SELECT ALL THAT APPLY) (READ OUT RESPONSE OPTIONS)

❑ Yes, from a friend (1)
❑ Yes, from a family member (2)
❑ Yes, from someone else (3). SPECIFY: ___________
❑ No (3)

CONDITION: IF USED TOBACCO IN THE LAST 30 days

The following questions ask about your intention to use tobacco in the next 12 months.

PROVIDE RESPONSE CARDS "A" TO ANSWER B17-B18 AND READ OUT RESPONSE OPTIONS FOR THE FIRST QUESTION AND AS NEEDED.

B17. At any time during the next 12 months, do you think you will smoke a cigarette or bidi?

❍ Definitely not (1)
❍ Probably not (2)
❍ Probably yes (3)
❍ Definitely yes (4)

B18. At any time during the next 12 months, do you think you will chew or apply tobacco in any form?

❍ Definitely not (1)
❍ Probably not (2)
❍ Probably yes (3)
❍ Definitely yes (4)

Please think about how hard or easy it is for you to do each of the following.

PROVIDE PARTICIPANTS WITH RESPONSE CARDS "B" TO HELP THEM ANSWER B19 – B21. READ OUT RESPONSE OPTIONS FOR THE FIRST TWO QUESTIONS AND AS NEEDED.

B19. Do you think it would be easy or hard for you to get tobacco products if you wanted them?

❍ Very easy (1)
❍ Somewhat easy (2)
❍ Somewhat hard (3)
❍ Very hard (4)

B20. If your friends offer you tobacco, would it be easy or hard for you to say no?

❍ Very easy (1)
❍ Somewhat easy (2)
❍ Somewhat hard (3)
❍ Very hard (4)

B21. If your relatives offer you tobacco, do you think it would be easy or hard for you to say no?

❍ Very easy (1)
❍ Somewhat easy (2)
❍ Somewhat hard (3)
❍ Very hard (4)

The next questions ask about your exposure to other people's tobacco use.

B22. During the past 7 days, on how many days has anyone smoked in your presence at home?

________ (DAYS: 0-7)

B23. During the past 7 days, on how many days has anyone smoked in your presence outside your home?

________ (DAYS: 0-7)

B24. During the past 7 days, on how many days has anybody smoked in your presence in enclosed public places? *(Public place include auditorium, hospitals, clinics, railway waiting room, amusement centers, restaurants, public offices, court buildings, educational institutions, libraries, bus and train stations and the like)*

________ (DAYS: 0-7)

B25. Have you seen <u>"No smoking" signs</u> in public places (such as in restaurants, stores, malls, buses, trains, schools, gyms, sport stadiums)

❍ Yes (1)
❍ No (2)

B26. Is tobacco in any form ever allowed inside your home? (READ OUT RESPONSE OPTIONS)

❍ Allowed (1)
❍ Allowed only at sometimes and in some places (2)
❍ Never allowed (3)

B27. During the past 12 months, how often did your parents communicate the following about tobacco use with you?

PROVIDE RESPONSE CARDS “C” TO PARTICIPANTS. READ OUT RESPONSE OPTIONS FOR FIRST QUESTION AND AS NEEDED.

a) Encourage you not to use tobacco	❍	❍	❍
b) Talk to you about the negative consequences of tobacco use	❍	❍	❍
c) Encourage you to choose friends who do not use tobacco	❍	❍	❍
d) Talk to you about the rules about tobacco use	❍	❍	❍

B28. How easy or hard is it for you to talk to your parents about tobacco use? (PROVIDE APRTICIPANTS WITH RESPONSE CARDS “B” TO HELP THEM ANSWER (OR) READ OUT RESPONSE OPTIONS)

- ❍ Very easy (1)
- ❍ Somewhat easy (2)
- ❍ Somewhat hard (3)
- ❍ Very hard (4)

The following questions are about your friends and peers.

B29. Think about your 5 closest friends. How many of them use tobacco?

________ (FRIENDS: 0-5)

B30. How many kids your age do you think use tobacco? (READ OUT RESPONSE OPTIONS)

❍ None of them (0)
❍ Some of them (1)
❍ Most of them (3)
❍ All of them (4)

B31. Does your school have a "No tobacco" policy?

❍ Yes (1)
❍ No (2)
❍ Don't know (3)

CONDITION: IF A3≠0.

B32. During the last school year, were you taught in any of your classes about the dangers of tobacco use?

❍ Yes (1)
❍ No (2)

CONDITION: IF A3≠0.

The next questions ask about going to stores and shops, and about tobacco products and advertisements in those places.

B33. In the past 30 days, how often did you go to the following?

PROVIDE PARTICIPANTS WITH RESPONSE CARDS "D". READ OUT RESPONSE OPTIONS FOR THE FIRST TWO QUESTIONS AND AS NEEDED.

	Never (1)	Monthly or less (2)	Once a week (3)	More than once a week (4)
a) General store	❍	❍	❍	❍
b) Small grocery store	❍	❍	❍	❍
c) Pan kiosk	❍	❍	❍	❍
d) Tea shop/stall	❍	❍	❍	❍
e) Street vendor	❍	❍	❍	❍

B34. How many stores and shops that sell tobacco are there within walking distance from your home? (*Stores include general stores, grocery stores, kiosks, pan kiosks, street shops, and so on)* (READ OUT RESPONSE OPTIONS)

- ❍ None (1)
- ❍ A few of them (2)
- ❍ A lot (3)

- ❍ **B35. In the past 30 days, how often did you see advertisements for tobacco products in the stores that sell tobacco within walking distance from home?** (PROVIDE PARTICIPANTS WITH RESPONSE CARDS "E" TO HELP THEM ANSWER AND READ OUT RESPONSE OPTIONS)
- ❍ Never (1)
- ❍ Rarely (2)
- ❍ Sometimes (3)
- ❍ Often (4)

B36. How many stores that sell tobacco are there within walking distance from your school? (*Stores include general stores, grocery stores, kiosks, pan kiosks, street shops, and so on)* (READ OUT RESPONSE OPTIONS)

- ❍ None (1)
- ❍ A few of them (2)
- ❍ A lot (3)

CONDITION: IF A3≠0.

B37. In the past 30 days, how often did you see advertisements for any tobacco products in the stores that sell tobacco within walking distance from school? (PROVIDE PARTICIPANTS WITH RESPONSE CARDS "E" TO HELP THEM ANSWER AND READ OUT RESPONSE OPTIONS)

- ❍ Never (1)
- ❍ Rarely (2)
- ❍ Sometimes (3)
- ❍ Often (4)

CONDITION: IF A3≠0.

The next questions ask about tobacco advertising and seeing tobacco use on TV, film and other places.

PROVIDE RESPONSE CARDS "F" TO PARTICIPANTS TO ANSWER E38 – E45. READ OUT RESPONSEOPTIONS THE FIRST TWO QUESTIONS AND AS NEEDED.

B38. During the past 30 days, how often did you see advertisements for cigarettes, bidis, gutka, pan masala or other tobacco products in <u>public transports</u> such as buses, autos, trains, taxis, etc.?

- ❍ Never (1)
- ❍ Sometimes (2)
- ❍ Often (3)

B39. How often did you see tobacco advertisements in <u>any media</u> such as television, newspapers, magazines, flyers, posters, hoardings?

- ❍ Never (1)
- ❍ Sometimes (2)
- ❍ Often (3)

B40. When you watch TV, videos, or movies, how often do you see actors <u>smoking</u>?

- ❍ Never (1)
- ❍ Sometimes (2)
- ❍ Often (3)
- ❍ I never watch TV, videos, or movies (4)

B41. When you watch TV, videos, or movies, how often do you see actors <u>chewing or applying tobacco</u>?

- ❍ Never (1)
- ❍ Sometimes (2)
- ❍ Often (3)
- ❍ I never watch TV, videos, or movies (4)

B42. During the past 30 days, when you watched sports events or other programs on TV, how often did you see brand names for cigarettes, bidis, gutka, pan masala or other tobacco products?

- ❍ Never (1)
- ❍ Sometimes (2)
- ❍ Often (3)
- ❍ I never watch TV, videos, or movies (4)

B43. When you go to sports events, fairs, concerts, community events, poojas, or weekly markets, how often do you see advertisements for cigarettes, bidis, gutka, pan masala or other tobacco products?

- ❍ Never (1)
- ❍ Sometimes (2)
- ❍ Often (3)
- ❍ I never attend such events (4)

B44. During the past 30 days, how often did you see or hear anti-tobacco messages in the media such as movies, TV, cinema theatres, radio?

❍ Never (1)
❍ Sometimes (2)
❍ Often (3)

B45. During the past 30 days, how often did you see or hear anti-tobacco messages in your neighborhood?

❍ Never (1)
❍ Sometimes (2)
❍ Often (3)

Section H: RELIGIOSITY

H1. What is your religion? (READ OUT RESPONSE OPTIONS)

❍ Hindu (1)
❍ Muslim (2)
❍ Christian (3)
❍ Sikh (4)
❍ Buddhist (5)
❍ Jain (6)
❍ Other (SPECIFY)_____________ (7)

H2. How often do you pray? (READ OUT RESPONSE OPTIONS)

❍ Never (1)
❍ 1-3 times a year (2)
❍ 1-3 times a month (3)
❍ 1-3 times a week (4)
❍ Nearly every day (5)

H3. How important is prayer at home to you? (READ OUT RESPONSE OPTIONS)

- ❍ Not at all important (1)
- ❍ Somewhat important (2)
- ❍ Very important (3)

H4. How often do you go to a place of worship (e.g., temple, mosque, church)? (READ OUT RESPONSE OPTIONS)

Never (1)

- ❍ 1-3 times a year (2)
- ❍ 1-3 times a month (3)
- ❍ 1-3 times a week (4)
- ❍ Nearly every day (5)

<u>Section I: MENTAL HEALTH</u>

The next few questions are about your feelings.

PROVIDE RESPONSE CARDS “H” TO PARTICPANTS TO HELP ANSWER I1 – I2. READ OUT RESPONSE OPTIONS FOR THE FIRST QUESTION AND AS NEEDED.

I1. During the past 30 days, how often have you felt lonely?

- ❍ Never (1)
- ❍ Sometimes (2)
- ❍ Most of the time (3)
- ❍ Always (4)

I2. During the past 30 days, how often have you been so worried about something that you could not sleep at night?

- ❍ Never (1)
- ❍ Sometimes (2)
- ❍ Most of the time (3)
- ❍ Always (4)

<u>Section J: FAMILY FACTORS</u>

Now, I'm going to ask a few questions about feelings and experiences in your relationship with your family.

J1. Please tell me how true the following statements are about your family:

PROVIDE RESPONSE CARDS "G" TO PARTICIPANTS, AND READ OUT RESPONSE OPTIONS FOR FIRST TWO QUESTIONS AND AS NEEDED.

	Very true (1)	A little true (2)	Not at all true (3)
a) Family members feel very close to each other	❍	❍	❍
b) We can easily think of things to do together as a family	❍	❍	❍
c) My family members ask each other for help	❍	❍	❍
d) I listen to what other family members have to say, even when I disagree	❍	❍	❍
e) My family members like to spend free time with me	❍	❍	❍
f) I am available when others in the family want to talk with me	❍	❍	❍

J2. Now, I'm going to ask a few questions about feelings and experiences that you have had about your <u>parent</u> or <u>main caregiver.</u> When you answer the following questions, please think of <NAME OF THE PARENT/MAIN CARETAKER>, who is completing the parent survey.

INFORM THE RESPONDENT THAT THE 'PARENT/MAIN CARETAKER' WE ARE TALKING ABOUT IS THE ONE WHO ANSWERS THE PARENT SURVEY.

Please tell me Yes or No to the following statements:

	Yes (1)	No (2)
a) My parent enjoys hearing what I say	❍	❍
b) I rely on my parent when I am upset or worried	❍	❍
c) My parent is good at helping me solve my problems	❍	❍

J3. Please tell me how often the following statements apply to you about how your parent or main care giver looks after you?

PROVIDE RESPONSE CARDS "F" TO PARTICIPANTS, AND READ OUT RESPONSE OPTIONS FOR FIRST TWO QUESTIONS AND AS NEEDED.

	Often (1)	Sometimes (2)	Never (3)
a) My parents tell me about when I'm supposed to be home after school	❍	❍	❍
b) How often is someone there after I come home from school	❍	❍	❍
c) I know how to contact my parents if they're away from me	❍	❍	❍
d) I always tell my parents when I go out with friends	❍	❍	❍

Section K: SCHOOL FACTORS

CONDITION: THE FOLLOWING QUESTIONS SHOULD BE ASKED ONLY TO THOSE WHO ARE CURRENTLY ATTENDING SCHOOL (A3≠0).

Now, I am going to ask few questions about your feelings and experiences in your school.

K1. Do you agree or disagree with each of the following statements?

PROVIDE RESPONSE CARDS "I" TO PARTICIPANTS, AND READ OUT RESPONSE OPTIONS FOR FIRST QUESTION AND AS NEEDED.

	Agree (1)	Neither Agree or disagree (2)	Disagree (3)
a) I feel close to my classmates at school	❍	❍	❍
b) I feel like I am part of school	❍	❍	❍
c) I'm happy to be at school	❍	❍	❍
d) I feel safe in my school	❍	❍	❍

K2. Is there a teacher or some other adult in your school who:

	Yes (1)	No (2)
a) Really cared about you	❍	❍
b) Told you when you do a good job	❍	❍
c) Noticed when you were not present	❍	❍
d) Always wanted you to do your best	❍	❍
e) Listened to you when you had something to say	❍	❍
f) Who believed in you	❍	❍
