# Supplementary material for: Religiosity, school connectedness, and tobacco use susceptibility: a longitudinal study of adolescents in Mumbai and Kolkata, India
Source: BMC Public Health. 2025 Nov 7;25:3857. doi: 10.1186/s12889-025-25035-7 (PMC12595895; doi:10.1186/s12889-025-25035-7)
Supplement: Supplementary file 2 — Supplementary Material 2. [file 12889_2025_25035_MOESM2_ESM.pdf]

Supplemental file #2-supplemental tables - results specific to combustible and smokeless tobacco use that parallel the "any tobacco use" reported in the text

Table S1a. Mumbai W1 and W1 to W2 change in adolescent tobacco use susceptibility (susceptibility to smoking, susceptibility to using smokeless and susceptibility to any form of tobacco) as a function of W1 & W1 to W2 change in respondent **school connectedness (3-item scale)**.

		Male			Female		
		Disagree or Neither agree nor disagree[c]	Agree	p-value for association[d]	Disagree or Neither agree nor disagree[c]	Agree	p-value for association[d]
Intention to smoke		n = 81	n = 336		n = 41	n = 358	
	W1[a]	18.9% (2.9, 34.8)	9.9% (4.4, 15.5)	.1152	0.8% (-0.8, 2.4)	3.5% (0.5, 6.6)	.2008
		n = 81	n = 333		n = 41	n = 352	
	W1-W2 change[b]	36.6% (21.1, 52.1)	22.6% (14.5, 30.7)	**.0379**	44.3% (25.2, 63.4)	20.1% (11.8, 28.4)	**.0010**
Intention to use smokeless		n = 82	n = 335		n = 41	n = 357	
	W1[a]	19.2% (3.9, 34.5)	7.8% (3.3, 12.4)	.0520	2.4% (-0.6, 5.4)	4.5% (1.4, 7.6)	.2883
		n = 82	n = 332		n = 41	n = 350	
	W1-W2 change[b]	39.4% (20.5, 58.2)	18.2% (9.1, 27.4)	.0368	29.0% (14.4, 43.6)	15.3% (8.0, 22.6)	.0350
Intent to smoke or use smokeless (combined)		n = 82	n = 336		41	358	
	W1[a]	20.0% (4.4, 35.6)	10.1% (4.6, 15.6)	.0725	2.4% (-0.6, 10.4)	3.9% (0.5, 5.4)	.1937
		n = 82	n = 333		n = 41	n = 353	
	W1-W2 change[b]	24.0% (15.1, 32.9)	46.5% (27.4,65.6)	**.0164**	20.7% (12.3, 29.0)	44.5% (25.4, 63.5)	**.0013**

a. Using W1 school connectedness information to predict W1 tobacco use susceptibility
b. Using W1-W2 change in school connectedness information to predict W1-W2 change in tobacco use susceptibility
c. These responses were in response to the question: "Do you feel part of your school ?– 1) Agree, 2) Neither agree nor disagree, 3) Disagree." For analysis purposes, this answer was reverse-scored so that agreement was associated increased school connectedness. Only 8 respondents chose the "Disagree" category, so these respondents were grouped with the respondents who chose the "Neither agree nor disagree" category.
d. These p-values were generated by weighted logistic regression (W1 religiosity predicting W1 tobacco use susceptibility or W1 religiosity predicting W1-W2 change in tobacco use susceptibility). Bolded values significant at $p < .05$. Respondent age was included as a covariate.

Table S1b. Kolkata W1 and W1 to W2 change in adolescent tobacco use susceptibility (susceptibility to smoking, susceptibility to using smokeless and susceptibility to any form of tobacco) as a function of W1 & W1 to W2 change in respondent **school connectedness (3-item scale)**.

		Male			Female		
		Disagree or Neither agree nor disagree[c]	Agree	p-value for association[d]	Disagree or Neither agree nor disagree[c]	Agree	p-value for association[d]
Intention to smoke		n = 140	n = 287		n = 133	n = 318	
	W1[a]	10.5% (-1.4, 22.5)	6.6% (0.8, 12.5)	.5798	1.9% (-0.5, 4.3)	8.5% (1.3, 15.6)	.0671
		n = 139	n = 284		n = 129	n = 316	
	W1-W2 change[b]	25.4% (12.1, 38.8)	18.3% (11.5, 25.0)	.3155	16.7% (5.0, 28.3)	17.3% (12.3, 22.3)	.9118
Intention to use smokeless		n = 140	n = 286		n = 133	n = 318	
	W1[a]	6.0% (0.6, 11.4)	5.9% (1.0, 10.7)	.9696	2.0% (-0.7, 4.8)	4.8% (0.9, 8.8)	.2574
		n = 139	n = 283		n = 129	n = 316	
	W1-W2 change[b]	23.3% (11.8, 34,7)	16.7% (9.2, 24.3)	.2844	14.4% (1.2, 27.1)	10.9% (5.8, 15.9)	.5065
Combined intent to smoke or use smokeless		n = 140	n = 287		n = 133	n = 318	
	W1[1]	10.5% (-1.5, 22.5)	8.7% (2.6, 14.8)	.8034	2.8% (-0.3, 5.9)	9.6% (2.4, 16.8)	.0735
		n = 139	n = 284		n = 129	n = 316	
	W1-W2 change[b]	28.4% (15.6, 41.3)	22.6% (16.1, 29.2)	.3903	19.3% (6.3, 32.3)	19.4% (13.5, 25.3)	.9854

a. Using W1 school connectedness information to predict W1 tobacco use susceptibility
b. Using W1-W2 change in school connectedness information to predict W1-W2 change in tobacco use susceptibility
c. These responses were in response to the question: "Do you feel part of your school ?– 1) Agree, 2) Neither agree nor disagree, 3) Disagree." For analysis purposes, this answer was reverse-scored so that agreement was associated increased school connectedness. Only 8 respondents chose the "Disagree" category, so these respondents were grouped with the respondents who chose the "Neither agree nor disagree" category.
d. These p-values were generated by weighted logistic regression (W1 religiosity predicting W1 tobacco use susceptibility) or by weighted logit mixed effects modeling (W1 religiosity predicting W2 tobacco use susceptibility). Bolded values represent p < .05. Respondent age was included as a covariate.

Table S2a. Mumbai W1 & W1-W2 change in adolescent tobacco use (susceptibility to smoking, susceptibility to using smokeless and combination of intent) as a function of W1 respondent **prayer frequency**.

		Male				Female			
		Praying 1-3 times per month or less[c]	Praying 1-3 times per week[c]	Praying nearly every day or more[c]	p-value for linear trend[d]	Praying 1-3 times per month or less[c]	Praying 1-3 times per week[c]	Praying nearly every day or more[c]	p-value for linear trend[d]
Intent to smoke within next year		n = 74	n = 157	n = 242		n = 65	n = 131	n = 263	
	W1[a]	26.5% (7.7, 45.4)	6.2% (0.1, 12.3)	11.5% (4.5, 18.6)	.0632	5.7% (-1.5, 12.8)	2.6% (-0.7, 5.9)	2.8% (0.6, 4.9)	.1683
		n = 64	n = 143	n = 214		n = 54	n = 119	n = 230	
	W1-W2 change[b]	24.5% (14.2, 34.7)	26.0% (11.9, 40.0)	24.9% (14,1, 35.8)	.9503	27.9% (9.8, 46.1)	19.1% (8.9, 29.3)	24.3% (11.5, 37.0)	.7368
Intent to use smoke-less within next year		n = 74	n = 157	n = 242		n = 64	n = 131	n = 263	
	W1[a]	25.4% (6.6, 44.2)	5.5% (0.0, 10.9)	8.0% (3.3 12.8)	**.0160**	5.7% (-1.5, 13.0)	2.3% (-0.8, 5.4)	4.6% (1.6, 7.7)	.7615
		n = 64	n = 143	n = 214		n = 53	n = 118	n = 230	
	W1-W2 change[b]	27.8% (13.2, 42.4)	22.1% (12.3, 31.9)	20.8% (10.2, 31.4)	.2510	29.3% (9.4, 49.1)	15.9% (6.1, 25.8)	15.8% (6.0, 25.5)	.2004
Intent to use any tobacco within next year		n = 74	n = 157	n = 243		n = 65	n = 131	n = 263	
	W1[a]	27.7% (8.9, 46.6)	6.8% (0.4, 13.2)	11.5% (4.5, 18.6)	.0446	5.7% (-1.5, 12.8)	2.9% (-0.5, 6.2)	5.0% (2.0, 8.1)	.8490
		n = 64	n = 143	n = 215		n = 54	n = 119	n = 230	
	W1-W2 change[b]	33.4% (21.4, 45.4)	28.4% (14.8, 42.0)	26.7% (15.4, 38.0)	.3540	28.7% (9.9, 47.5)	19.3% (9.0, 29.6)	25.2% (12.9, 37.5)	.7491

a. Using W1 religiosity information to predict W1 tobacco use susceptibility
b. Using W1 school connectedness information to predict W1-W2 change in tobacco use susceptibility
c. These responses were in response to the question: "How often do you pray?" Answer options included: 1) I never pray, 2) I pray 1-3 times per year, 3) I pray 1-3 times per month, 4) I pray 1-3 times per week, 5) I pray nearly every day or more. To normalize the resulting skewed distribution, for analysis purposes answer options 1, 2, and 3 were combined into “Praying 1-3 times per month or less.”
d. These p-values were generated by weighted logistic regression (W1 religiosity predicting W1 tobacco use susceptibility) or by weighted logit mixed effects modeling (W1 religiosity predicting W2 tobacco use susceptibility). Bolded values represent p < .05. Respondent age was included as a covariate.

Table S2b. Kolkata W1 & W1-W2 change in adolescent tobacco use (susceptibility to smoking, susceptibility to using smokeless and combination of intent) as a function of W1 respondent **prayer frequency**.

		Male				Female			
		Praying 1-3 times per month or less[c]	Praying 1-3 times per week[c]	Praying nearly every day or more[c]	p-value for linear trend[d]	Praying 1-3 times per month or less[c]	Praying 1-3 times per week[c]	Praying nearly every day or more[c]	p-value for linear trend[d]
Intent to smoke within next year		n = 194	n = 151	n = 156		n = 139	n = 164	n = 223	
	W1[a]	6.4% (-1.1, 13.9)	11.4% (2.2, 20.6)	8.4% (-1.6, 18.4)	.7580	5.8% (-0.2, 11.8)	6.1% (-1.8, 13.9)	7.1% (-1.3, 15.4)	.8100
		n = 162	n = 138	n = 138		n = 121	n = 144	n = 193	
	W1-W2 change[b]	23.9% (13.6, 34.2)	22.1% (12,7, 31.5)	15.5% (4.8, 26.1)	.2632	18.8% (10.1, 27.4)	12.2% (7.1, 17.3)	18.4% (10.9, 25.8)	.9273
Intent to use smoke-less within next year		n = 194	n = 151	n = 155		n = 139	n = 164	n = 223	
	W1[a]	5.6% (0.8, 10.4)	8.6% (0.5, 16.7)	4.8% (1.8, 7.7)	.7428	2.4% (-1.3, 6.1)	2.4% (-2.1, 6.9)	5.7% (-0.9, 12.3)	.4078
		n = 162	n = 138	n = 137		n = 121	n = 144	n = 193	
	W1-W2 change[b]	22.3% (12.9, 31.6)	17.7% (6.0, 29.5)	15.3% (5.1, 25.4)	.3044	10.4% (2.6, 18.1)	11.6% (3.0, 20.2)	13.0% (6.3, 19.6)	.5009
Intent to use any tobacco within next year		n = 194	n = 151	n = 156		n = 139	n = 164	n = 223	
	W1[a]	8.4% (0.4, 16.5)	10.9% (1.8, 19.9)	10.3% (0.1, 20.4)	.7908	5.6% (-0.4, 11.6)	8.5% (-0.3, 17.2)	7.4% (-0.7, 15.6)	.7358
		n = 162	n = 138	n = 138		n = 121	n = 144	n = 193	
	W1-W2 change[b]	28.3% (18.2, 38.3)	25.6% (15.4, 35.7)	18.8% (9.4, 28.2)	.6207	19.1% (10.2, 27.9)	17.3% (10.3, 24.4)	19.9% (12.4, 27.3)	.7298

a. Using W1 religiosity information to predict W1 tobacco use susceptibility
b. Using W1 school connectedness information to predict W1-W2 change in tobacco use susceptibility
c. These responses were in response to the question: "How often do you pray?" Answer options included: 1) I never pray, 2) I pray 1-3 times per year, 3) I pray 1-3 times per month, 4) I pray 1-3 times per week, 5) I pray nearly every day or more. To normalize the resulting skewed distribution, for analysis purposes answer options 1, 2, and 3 were combined into “Praying 1-3 times per month or less.”
d. These p-values were generated by weighted logistic regression (W1 religiosity predicting W1 tobacco use susceptibility) or by weighted logit mixed effects modeling (W1 religiosity predicting W2 tobacco use susceptibility). Bolded values represent $p < .05$. Respondent age was included as a covariate.

Table S3a. Mumbai W1 & W1-W2 change in adolescent tobacco use (susceptibility to smoking, susceptibility to using smokeless and combination of intent) as a function of the respondent's W1 rated **importance of prayer.** Covariate = age.

		Male				Female			
		Prayer not at all important[c]	Prayer somewhat important[c]	Prayer very important[c]	p-value for linear trend[d]	Prayer not at all important[c]	Prayer somewhat important[c]	Prayer very important[c]	p-value for linear trend[d]
Intent to smoke within next year		n = 15	n = 124	n = 333		n = 12	n = 87	n = 358	
	W1[a]	46.1% (23.8, 68.4)	15.1% (2.1, 28.1)	9.2% (3.4, 15.0)	**.0001**	6.2% (-4.9, 17.2)	4.1% (0.7, 7.6)	2.9% (0.3, 5.6)	.3112
		n = 14	n = 108	n = 298		n = 11	n = 79	n = 311	
	W1-W2 change[b]	37.9% (26.9, 48.9)	25.7% (17.2, 34.3)	23.9% (13.0, 34.8)	.1180	14.9% (-6.9, 36.6)	20.2% (6.0, 34.4)	23.9% (13.9, 33.8)	.3859
Intent to use smoke-less within next year		n = 15	n = 124	n = 333		n = 12	n = 87	n = 357	
	W1[a]	51.3% (31.4, 71.1)	12.1% (1.8, 22.4)	7.0% (2.5, 11.5)	**<.0001**	15.7% (-12.5, 43.8)	4.8% (-1.0, 10.6)	3.8% (1.1, 6.4)	.1157
		n = 14	n = 108	n = 298		n = 11	n = 79	n = 309	
	W1-W2 change[b]	48.5% (22.2, 74.8)	24.4% (13.8, 35.0)	19.7% (10.2, 29.2)	**.0021**	15.0% (-7.4, 37.5)	20.1% (5.9, 34.3)	16.7% (9.0, 24.4)	.8589
Intent to use any tobacco within next year		n = 15	n = 124	n = 334		n = 12	n = 87	n = 358	
	W1[a]	51.6% (32.1, 71.2)	16.2% (3.0, 29.5)	9.0% (3.4, 14.7)	**<.0001**	15.6% (-12.5, 43.7)	5.6% (-0.4, 11.6)	4.0% (1.2, 6.8)	.1202
		n = 14	n = 108	n = 299		n = 11	n = 79	n = 311	
	W1-W2 change[b]	51.4% (28.0, 74.8)	31.2% (21.9, 40.5)	25.6% (14.6, 36.5)	**.0059**	14.9% (-7.0, 36.9)	20.3% (6.2, 34.5)	24.7% (14.9, 34.4)	.3267

a. Using W1 religiosity information to predict W1 tobacco use susceptibility
b. Using W1 school connectedness information to predict W1-W2 change in tobacco use susceptibility
c. These responses were in response to the question: "How important is prayer at home to you?" Answer options were: 1) Prayer is not all important to me, 2) Prayer is somewhat important to me, 3) Prayer is very important to me.
d. These p-values were generated by weighted logistic regression (W1 religiosity predicting W1 tobacco use susceptibility) or by weighted logit mixed effects modeling (W1 religiosity predicting W2 tobacco use susceptibility). Bolded values exceed $p < .05$. Respondent age was included as a covariate.

Table S3b. Kolkata W1 & W1-W2 change in adolescent tobacco use (susceptibility to smoking, susceptibility to using smokeless and combination of intent) as a function of the respondent's rated W1 **importance of prayer.** Covariate = age.

		Male				Female			
		Prayer not at all important[c]	Prayer somewhat important[c]	Prayer very important[c]	p-value for linear trend[d]	Prayer not at all important[c]	Prayer somewhat important[c]	Prayer very important[c]	p-value for linear trend[d]
Intent to smoke within next year			n = 216	n = 284			n = 184	n = 343	
	W1[a]	--------	9.4% (2.1, 16.8)	6.7% (1.2, 12.2)	.5701	--------	10.5% (-1.4, 22.4)	4.1% (0.8, 7.4)	.1721
			n = 191	n = 245			n = 171	n = 288	
	W1-W2 change[b]	--------	20.7% (11.0, 30.4)	21.2% (11.3, 31.2)	.9365	--------	21.8% (12.5, 31.1)	13.3% (8.8, 17.8)	.0129
Intent to use smoke-less within next year			n = 216	n = 283			n = 184	n = 343	
	W1[a]	--------	8.6% (3.8, 13.3)	4.0% (1.0, 6.9)	.1192	--------	8.2% (0.8, 15.6)	1.1% (0.0, 2.2)	**.0028**
			n = 192	n = 244			n = 171	n = 288	
	W1-W2 change[b]	--------	19.1% (7.3, 31.0)	18.9% (9.1, 28.8)	.9796	--------	16.0% (5.7, 26.2)	8.8% (3.7, 14.0)	**.0350**
Intent to use any tobacco within next year			n = 216	n = 284			n = 184	n = 343	
	W1[a]	--------	11.4% (4.5, 18.5)	7.6% (1.7, 13.5)	.4207	--------	12.9% (1.2, 24.7)	4.2% (1.0, 7.5)	.0504
			n = 192	n = 245			n = 171	n = 288	
	W1-W2 change[b]	--------	25.2% (13.5, 37.0)	24.5% (15.8, 33.2)	.9213	--------	25.2% (15.0, 35.4)	14.7% (9.8, 19.6)	**.0028**

a. Using W1 religiosity information to predict W1 tobacco use susceptibility
b. Using W1 school connectedness information to predict W1-W2 change in tobacco use susceptibility
c. These responses were in response to the question: "How important is prayer at home to you?" Answer options were: 1) Prayer is not all important to me, 2) Prayer is somewhat important to me, 3) Prayer is very important to me. Only one Kolkata respondent selected "Not at all important," so this value was included in the "Prayer is somewhat important" category for analysis purposes.
d. These p-values were generated by weighted logistic regression (W1 religiosity predicting W1 tobacco use susceptibility) or by weighted logit mixed effects modeling (W1 religiosity predicting W2 tobacco use susceptibility). Bolded values exceed $p < .05$. Respondent age was included as a covariate.

Table S4a. Mumbai W1 & W1-W2 change in adolescent tobacco use (susceptibility to smoking, susceptibility to using smokeless and combination of intent) as a function of the respondent's W1 **frequency of attendance at a temple, mosque**, etc. Covariate = age.

		Male						Female					
		Never attends temple	Attends temple 1 to 3 times per year	Attends temple 1 to 3 times per month	Attends temple 1 to 3 times per week	Attends temple nearly every day	p-value for linear trend[d]	Never attends temple	Attends temple 1 to 3 times per year	Attends temple 1 to 3 times per month	Attends temple 1 to 3 times per week	Attends temple nearly every day	p-value for linear trend[d]
Intent to smoke within next year		n = 64	n = 64	n = 168	n = 123	n = 52		n = 48	n = 75	n = 142	n = 141	n = 53	
	W1[a]	13.6% (-1.9, 29.0)	16.2% (2.2, 30.2)	8.1% (2.0, 14.2)	14.0% (4.3, 23.7)	14.8% (-3.3, 33.0)	.9893	2.5% (-1.0, 5.9)	6.3% (-2.2, 14.7)	3.5% (-0.3, 7.2)	1.6% (-0.7, 3.8)	2.8% (-1.7, 7.3)	.5595
		n = 61	n = 52	n = 154	n = 106	n = 46		n = 41	n = 57	n = 123	n = 133	n = 49	
	W1-W2 diff[b]	25.9% (16.2, 35.6)	43.8% (22.7, 64.9)	19.7% (13.1, 26.3)	30.0% (12.0, 48.0)	13.0% (-2.6, 28.6)	.0647	34.5% (15.2, 53.9)	28.6% (18.3, 38.9)	16.7% (4.5, 28.9)	22.1% (11.1, 33.2)	19.9% (3.5, 36.3)	.1511
Intent to use smoke-less within next year		n = 64	n = 64	n = 168	n = 123	n = 52		n = 48	n = 75	n = 142	n = 141	n = 52	
	W1[a]	7.9% (-2.9, 18.8)	14.3% (0.4, 28.1)	6.9% (1.1, 12.7)	12.4% (3.8, 21.0)	11.6% (-4.0, 27.2)	.7732	2.5% (-1.0, 5.9)	10.0% (2.0, 17.9)	4.5% (-0.2, 9.1)	1.6% (-0.3, 3.5)	2.9% (-1.7, 7.6)	.3436
		n = 61	n = 52	n = 154	n = 106	n = 46		n = 41	n = 56	n = 123	n = 133	n = 48	
	W1-W2 diff[b]	24.1% (15.5, 32.7)	35.3% (18.0, 52.7)	16.7% (7.3, 26.1)	31.1% (13.9, 48.4)	6.8% (0.5, 13.1)	**.0031**	25.9% (12.4, 39.4)	22.0% (12.7, 39.4)	13.9% (2.9, 25.0)	16.5% (8.8, 24.3)	11.3% (1.8, 20.9)	.0582
Intent to use any tobacco within next year		n = 64	n = 64	n = 169	n = 123	n = 52		n = 48	n = 75	n = 142	n = 141	n = 53	
	W1[a]	13.5% (-1.9, 29.0)	16.3% (2.0, 30.6)	8.3% (2.0, 14.6)	14.4% (4.8, 23.9)	16.5% (-1.2, 34.3)	.8671	2.5% (-1.0, 5.9)	10.0% (2.1, 18.0)	4.8% (0.0, 9.6)	2.5% (0.0, 4.9)	2.9% (-1.7, 7.5)	.3369
		n = 61	n = 52	n = 155	n = 106	n = 46		n = 41	n = 57	n = 123	n = 133	n = 49	
	W1-W2 diff[b]	31.4% (21.7, 41.2)	45.6% (25.0, 66.3)	22.4% (13.6, 31.2)	32.6% (14.6, 50.7)	15.9% 0.3, 31.5)	**.0185**	34.8% (15.3, 54.2)	30.2% (18.4, 41.9)	17.9% (5.3, 30.5)	22.2% (11.2, 33.2)	20.1% (3.7, 36.6)	.1398

a. Using W1 religiosity information to predict W1 tobacco use susceptibility
b. Using W1 school connectedness information to predict W1-W2 change in tobacco use susceptibility
c. Responses were answers to the question: "How often do you go to a place of worship(e.g. temple, mosque or church)?"
d. These p-values were generated by weighted logistic regression (W1 religiosity predicting W1 tobacco use susceptibility) or by weighted logit mixed effects modeling (W1 religiosity predicting W2 tobacco use susceptibility). Bolded values exceed $p < .05$. Respondent age was included as a covariate.

Table S4b. Kolkata W1 and W1-W2 change in adolescent tobacco use (susceptibility to smoking, susceptibility to using smokeless and combination of intent) as a function of the respondent's W1 **frequency of attendance at a temple, mosque**, etc. Covariate = age.

		Male						Female					
		Never attends temple	Attends temple 1 to 3 times per year	Attends temple 1 to 3 times per month	Attends temple 1 to 3 times per week	Attends temple nearly every day	p-value for linear trend[d]	Never attends temple	Attends temple 1 to 3 times per year	Attends temple 1 to 3 times per month	Attends temple 1 to 3 times per week	Attends temple nearly every day	p-value for linear trend[d]
Intent to smoke within next year		n = 112	n = 121	n = 145	n = 87	n = 34		n = 113	n = 120	n = 187	n = 69	n = 36	
	W1[a]	4.0% (0.3, 7.7)	4.5% (1.0, 8.0)	10.9% (2.4, 19.4)	14.2% (-9.0, 37.4)	10.9% (-2.4, 24.2)	.1949	8.0% (-2.3, 18.3)	7.7% (-0.9, 37.4)	5.0% (-1.0, 11.0)	6.6% (-2.9, 16.1)	3.0% (-1.2, 7.2)	.4407
		n = 94	n = 103	n = 125	n = 82	n = 33		n = 93	n = 102	n = 169	n = 59	n = 35	
	W1-W2 diff[b]	13.4% (1.2, 25.6)	23.5% (13.8, 33.2)	21.1% (12.2, 30.0)	24.1% (9.1, 39.2)	23.6% (7.0, 40.1)	.2933	16.9% (5.2, 28.5)	19.0% (8.6, 29.5)	13.1% (6.0, 20.1)	10.0% (-2.6, 22.6)	30.8% (12.6, 49.0)	.2869
Intent to use smoke -less within next year		n = 112	n = 121	n = 144	n = 87	n = 34		n = 113	n = 120	n = 187	n = 69	n = 36	
	W1[a]	4.6% (0.6, 8.7)	6.5% (1.3, 11.8)	6.8% (2.0, 11.6)	1.6% (-1.3, 4.5)	15.9% (0.4, 31.4)	.2914	7.5% (-3.6, 18.7)	5.2% (-1.3, 11.8)	0.4% (0.0, 0.8)	7.1% (-3.9, 18.1)	1.2% (-1.3, 3.7)	.4502
		n = 94	n = 103	n = 124	n = 82	n = 33		n = 93	n = 102	n = 169	n = 59	n = 35	
	W1-W2 diff[b]	16.2% (2.2, 30.2)	20.6% (6.7, 34.5)	16.2% (6.1, 26.3)	25.4% (9.2, 41.5)	20.3% (5.9, 34.7)	.5227	12.2% (-0.1, 24.6)	9.4% (3.1, 15.7)	12.4% (5.5, 19.3)	6.1% (-5.8, 18.0)	19.0% (-1.9, 40.0)	.5719
Intent to use any tobacc o within next year		n = 112	n = 121	n = 145	n = 87	n = 34		n = 113	n = 120	n = 187	n =69	n = 36	
	W1[a]	5.7% (2.0, 9.3)	7.6% (2.0, 13.2)	10.8% (2.3, 19.3)	14.4% (-9.5, 38.3)	16.0% (2.4, 29.5)	.1500	9.5% (-0.5, 19.5)	9.8% (1.1, 18.4)	5.0% (-1.0, 11.0)	6.8% (-3.1, 16.6)	3.1% (-1.3, 7.5)	.2646
		n = 94	n = 103	n = 125	n = 82	n = 33		n = 93	n = 102	n = 169	n = 59	n = 35	
	W1-W2 diff[b]	19.0% (7.8, 30.2)	27.0% (14.0, 40.0)	23.0% (13.5, 32.5)	30.6% (13.9, 47.2)	27.5% (12.4, 42.6)	.3471	20.7% (10.2, 31.2)	19.7% (9.4, 29.9)	16.4% (8.3, 24.6)	10.1% (-2.7, 23.0)	31.4% (12.9, 49.8)	.9045

a. Using W1 religiosity information to predict W1 tobacco use susceptibility
b. Using W1 school connectedness information to predict W1-W2 change in tobacco use susceptibility
c. Responses were answers to the question: "How often do you go to a place of worship(e.g. temple, mosque or church)?"
e. These p-values were generated by weighted logistic regression (W1 religiosity predicting W1 tobacco use susceptibility) or by weighted logit mixed effects modeling (W1 religiosity predicting W2 tobacco use susceptibility). Bolded values exceed $p < .05$. Respondent age was included as a covariate.
