# Supplementary material for: Religiosity, school connectedness, and tobacco use susceptibility: a longitudinal study of adolescents in Mumbai and Kolkata, India
Source: BMC Public Health. 2025 Nov 7;25:3857. doi: 10.1186/s12889-025-25035-7 (PMC12595895; doi:10.1186/s12889-025-25035-7)
Supplement: Supplementary file 3 — Supplementary Material 3. [file 12889_2025_25035_MOESM3_ESM.pdf]

Supplemental file no. 3 -The regression statistics associated with the text results

Regression statistics for Mumbai, W1 predictors and Wave 1 tobacco use susceptibility:

Table S5a. Mumbai W1 school connectedness as a predictor of W1 tobacco use susceptibility.

**Outcome**: W1 Tobacco use susceptibility	Odds Ratio	p-value*	Lower bound of 95% CI	Upper bound of 95% CI
**Predictors**:				
Gender	0.04	**.006**	0.00	0.38
School connectedness				
Ambivalent about feeling connected to school	Ref.			
Fully agree that they feel well-connected to their school	0.29	**.015**	0.11	0.78
Gender x School connectedness				
Female x Fully agree	9.81	.083	0.73	131.78
Age	0.91	.650	0.59	1.39

*note. Wave 1 sampling weights used; p-values < .05 are bolded.

Table S5b. Mumbai W1 prayer frequency as a predictor of W1 tobacco use susceptibility.

**Outcome**: W1 Tobacco use susceptibility	Odds Ratio	p-value	Lower bound of 95% CI	Upper bound of 95% CI
**Predictors**:				
Gender	0.16	**.019**	0.04	0.73
Prayer frequency				
1-3 times per month or less	Ref.			
1-3 times per week	0.19	**.009**	0.06	0.64
Nearly every day	0.34	**.045**	0.12	0.97
Gender x Prayer frequency				
Female x 1-3 times per week	2.58	0.42	0.25	26.39
Female x Nearly every day	2.60	0.26	0.49	13.85
Age	0.96	0.81	0.65	1.40

*note. Wave 1 sampling weights used; p-values < .05 are bolded.

Table S5c. Mumbai W1 prayer importance as a predictor of W1 tobacco use susceptibility.

**Outcome**: W1 Tobacco use susceptibility	Odds Ratio	p-value	Lower bound of 95% CI	Upper bound of 95% CI
**Predictors**:				
Gender	0.17	.08	0.02	1.27
Prayer Importance				
Not at all important	Ref.			

Somewhat important	0.18	**.003**	0.06	0.54
Very important	0.09	**.000**	0.04	0.21
Gender x Prayer frequency				
Female x Somewhat important	1.77	.43	0.67	25.52
Female x Very important	2.46	.88	0.38	19.13
Age	0.84	.81	0.56	1.26

*note. Wave 1 sampling weights used; p-values < .05 are bolded.

Table S5d. Mumbai W1 frequency of attendance at a place of worship as a predictor of W1 tobacco use susceptibility.

**Outcome**: W1 Tobacco use susceptibility	Odds Ratio	p-value	Lower bound of 95% CI	Upper bound of 95% CI
**Predictors**:				
Gender	0.16	.07	0.02	1.15
Frequency of attendance at house of worship				
Almost never	Ref.			
1-3 times per year	1.24	.78	0.26	6.03
1-3 times per month	0.58	.45	0.14	2.46
1-3 times per week	1.07	.93	0.23	5.03
Nearly every day	1.26	.81	0.19	8.58
Gender x Prayer frequency				
Female x 1-3 times per year	3.56	.28	0.35	36.37
Female x 1-3 times per month	3.48	.16	0.59	20.52
Female x 1-3 times per week	0.94	.94	0.15	6.00
Female x Nearly every day	0.93	.96	0.06	15.12
Age	0.92	.64	0.65	1.30

*note. Wave 1 sampling weights used

Regression statistics for Kolkata, W1 predictors and Wave 1 tobacco use susceptibility:

Table S5e. Kolkata W1 school connectedness as a predictor of W1 tobacco use susceptibility.

**Outcome**: W1 Tobacco use susceptibility	Odds Ratio	p-value	Lower bound of 95% CI	Upper bound of 95% CI
**Predictors**:				
Gender	0.11	.094	0.01	1.48
School connectedness				
Ambivalent about feeling connected to school	Ref.			
Fully agree that they feel well-connected to their school	0.44	.390	.067	2.93

Gender x School connectedness				
Female x Fully agree	8.55	.117	0.57	127.90
Age	1.23	.469	0.69	2.18

*note. Wave 1 sampling weights used

Table S5f. Kolkata W1 prayer frequency as a predictor of W1 tobacco use susceptibility.

**Outcome**: W1 Tobacco use susceptibility	Odds Ratio	p-value	Lower bound of 95% CI	Upper bound of 95% CI
**Predictors**:				
Gender	0.64	.590	0.12	3.31
Prayer frequency				
1-3 times per month or less	Ref.			
1-3 times per week	1.32	.732	0.26	6.54
Nearly every day	1.23	.791	0.25	6.00
Gender x Prayer frequency				
Female x 1-3 times per week	1.18	.879	0.13	10.56
Female x Nearly every day	1.09	.942	0.09	13.17
Age	1.27	.441	0.68	2.37

*note. Wave 1 sampling weights used

Table S5g. Kolkata W1 prayer importance as a predictor of W1 tobacco use susceptibility.

**Outcome**: W1 Tobacco use susceptibility	Odds Ratio	p-value	Lower bound of 95% CI	Upper bound of 95% CI
**Predictors**:				
Gender	1.15	.803	0.38	3.44
Prayer Importance				
Not at all /somewhat important	Ref			
Very important	0.63	.421	0.20	1.98
Gender x Prayer frequency				
Female x Very important	0.47	.273	0.12	1.85
Age	1.21	.495	0.69	2.10

*note. Wave 1 sampling weights used

Table S5h. Kolkata W1 frequency of attendance at a place of worship as a predictor of W1 tobacco use susceptibility.

**Outcome**: W1 Tobacco use susceptibility	Odds Ratio	p-value	Lower bound of 95% CI	Upper bound of 95% CI
**Predictors**:				
Gender	2.08	.332	0.46	9.43

Frequency of attendance at house of worship				
Almost never	Ref.			
1-3 times per year	1.13	.860	0.27	4.71
1-3 times per month	2.94	.069	0.92	9.41
1-3 times per week	3.99	.221	0.42	37.63
Nearly every day	2.95	.217	0.52	16.78
Gender x Prayer frequency				
Female x 1-3 times per year	0.85	.896	0.07	10.20
Female x 1-3 times per month	0.21	.174	0.02	2.06
Female x 1-3 times per week	0.20	.330	0.01	5.25
Female x Nearly every day	0.12	.087	0.01	1.38
Age	1.45	.112	0.91	2.30

*note. Wave 1 sampling weights used

---

*note. now focusing on W1 predictors of W1-W2 change in tobacco use intention

Regression statistics for Mumbai Wave 1 predictors of W2 tobacco use intention:

Table S6a. Mumbai W1 school connectedness as a predictor of W1-W2 change in tobacco use susceptibility.

**Outcome**: W1-W2 Change in tobacco use susceptibility	Odds Ratio	p-value	Lower bound of 95% CI	Upper bound of 95% CI
**Predictors**:				
Gender	0.59	0.511	0.12	2.94
W1 school connectedness				
Ambivalent about feeling connected to school	Ref.			
Agree that they feel well-connected to their school	0.40	**.024**	0.18	0.88
Gender x School connectedness				
Female x Fully agree	1.42	0.658	0.29	6.80
Age	0.86	0.349	0.62	1.18

*note. Wave 2 sampling weights used; p-values < .05 are bolded.

Table S6b. Mumbai W1 prayer frequency as a predictor of W1-W2 change in tobacco use susceptibility.

**Outcome**: W1-W2 Change in tobacco use susceptibility	Odds Ratio	p-value	Lower bound of 95% CI	Upper bound of 95% CI
**Predictors**:				
Gender	0.80	.649	0.30	2.13
W1 Prayer frequency				
1-3 times per month or less	Ref.			
1-3 times per week	0.79	.207	0.54	1.14
Nearly every day	0.73	.354	0.36	1.45
Gender x Prayer frequency				
Female x 1-3 times per week	0.75	.559	0.28	1.99
Female x Nearly every day	1.15	.417	0.63	4.56
Age	0.88	.468	0.64	1.21

*note. Wave 2 sampling weights used

Table S6c. Mumbai W1 prayer frequency as a predictor of W1-W2 change in tobacco use susceptibility.

**Outcome**: W1-W2 Change in tobacco use susceptibility	Odds Ratio	p-value	Lower bound of 95% CI	Upper bound of 95% CI
**Predictors**:				
Gender	0.17	**.003**	0.05	0.52
W1 Prayer Importance				
Not at all important	Ref.			
Somewhat important	0.43	**.050**	0.18	1.00
Very important	0.32	**.006**	0.15	0.71
Gender x Prayer frequency				
Female x Somewhat important	3.41	**.030**	1.13	10.26
Female x Very important	5.78	**.008**	1.64	20.40
Age	0.84	.280	0.61	1.16

*note. Wave 2 sampling weights used; p-values < .05 are bolded.

Table S6d. Mumbai W1 frequency of attendance at a place of worship as a predictor of W1-W2 change in tobacco use susceptibility.

**Outcome**: W1-W2 Change in tobacco use susceptibility	Odds Ratio	p-value	Lower bound of 95% CI	Upper bound of 95% CI
**Predictors**:				
Gender	1.16	.693	0.54	2.50
W1 Frequency of attendance at house of worship				
Almost never	Ref.			
1-3 times per year	1.83	.190	0.73	4.58
1-3 times per month	0.63	.067	0.38	1.04
1-3 times per week	1.06	.894	0.45	2.48
Nearly every day	0.41	.158	0.12	1.43

Gender x Prayer frequency				
Female x 1-3 times per year	0.44	.350	0.08	2.52
Female x 1-3 times per month	0.65	.126	0.37	1.13
Female x 1-3 times per week	0.51	.171	0.19	1.36
Female x Nearly every day	1.15	.821	0.34	3.86
Age	0.88	.368	0.65	1.18

*note. Wave 2 sampling weights used

Regression statistics for Mumbai – Association of W1-W2 change in school connectedness with W1-W2 change in tobacco use susceptibility.

Table S7a. Mumbai W1-W2 change in school connectedness as a predictor of W1-W2 change in tobacco use susceptibility.

**Outcome**: W1-W2 Change in tobacco use susceptibility	Odds Ratio	p-value*	Lower bound of 95% CI	Upper bound of 95% CI
**Predictors**:				
Gender	0.92	0.877	0.32	2.68
Change in school connectedness				
Ambivalent about feeling connected to school	Ref.			
Consistently agree that they feel well-connected to their school	0.36	**.016**	0.16	0.82
Gender x School connectedness				
Female x Fully agree	0.89	.838	0.30	2.68
Age	0.85	.356	0.61	1.20

*note. Wave 2 sampling weights used; p-values < .05 are bolded.

Regression statistics for Kolkata Wave 1 predictors and W1 to W2 change in tobacco use susceptibility:

Table S6e. Kolkata W1 school connectedness as a predictor of W1-W2 change in tobacco use susceptibility.

**Outcome**: W1-W2 Change in tobacco use susceptibility	Odds Ratio	p-value	Lower bound of 95% CI	Upper bound of 95% CI
**Predictors**:				
Gender	0.13	**.002**	0.04	0.46
W1 school connectedness				

Ambivalent about feeling connected to school	Ref.			
Agree that they feel well-connected to their school	0.77	.450	0.40	1.55
Gender x School connectedness				
Female x Fully agree	6.54	**.006**	1.77	24.10
Age	1.28	.118	0.94	1.75

*note. Wave 2 sampling weights used; p-values < .05 are bolded.

Table S6f. Kolkata W1 prayer frequency as a predictor of W1-W2 change in tobacco use susceptibility.

**Outcome**:<br>W1-W2 Change in tobacco use susceptibility	Odds Ratio	p-value	Lower bound of 95% CI	Upper bound of 95% CI
**Predictors**:				
Gender	0.59	.233	0.25	1.41
W1 Prayer frequency				
1-3 times per month or less	Ref.			
1-3 times per week	0.87	.621	0.49	1.53
Nearly every day	0.58	.181	0.26	1.29
Gender x Prayer frequency				
Female x 1-3 times per week	1.02	.959	0.43	2.41
Female x Nearly every day	1.80	.191	0.74	4.40
Age	1.34	.112	0.93	1.92

*note. Wave 2 sampling weights used

Table S6g. Kolkata W1 prayer importance as a predictor of W1-W2 change in tobacco use susceptibility.

**Outcome**:<br>W1-W2 Change in tobacco use susceptibility	Odds Ratio	p-value	Lower bound of 95% CI	Upper bound of 95% CI
**Predictors**:				
Gender	1.00	.999	0.37	2.70
W1 Prayer Importance				
Not at all /somewhat important	Ref.			
Very important	0.96	.921	0.42	2.20
Gender x Prayer frequency				
Female x Very important	0.53	.207	0.20	1.44
Age	1.29	.100	0.95	1.75

*note. Wave 2 sampling weights used

Table S6h. Kolkata W1 frequency of attendance as a predictor of W1-W2 change in tobacco use susceptibility.

**Outcome**:<br>W1-W2 Change in tobacco use susceptibility	Odds Ratio	p-value	Lower bound of 95% CI	Upper bound of 95% CI

Predictors:				
Gender	1.11	.822	0.42	2.94
W1 Frequency of attendance at house of worship				
Almost never	Ref.			
1-3 times per year	1.59	.349	0.59	4.23
1-3 times per month	1.28	.571	0.54	3.05
1-3 times per week	1.89	.185	0.73	4.88
Nearly every day	1.62	.409	0.50	5.23
Gender x Prayer frequency				
Female x 1-3 times per year	0.59	.387	0.18	1.98
Female x 1-3 times per month	0.59	.289	0.22	1.60
Female x 1-3 times per week	0.23	.160	0.03	1.83
Female x Nearly every day	1.08	.907	0.28	4.25
Age	1.30	.104	0.95	1.78

*note. Wave 2 sampling weights used

Regression statistics for W1-W2 change in Kolkata school connectedness and W1-W2 change in tobacco use intention:

Table S7b. Kolkata W1-W2 change in school connectedness as a predictor of W1-W2 change in tobacco use susceptibility.

**Outcome**: W1-W2 Change in tobacco use susceptibility	Odds Ratio	p-value	Lower bound of 95% CI	Upper bound of 95% CI
**Predictors**:				
Gender	0.60	.435	0.16	2.22
W1-W2 Change in School connectedness				
Ambivalent about feeling connected to school	Ref.			
Consistently agree that they feel well-connected to their school	0.73	.390	0.36	1.51
Gender x School connectedness				
Female x Fully agree	1.37	.615	0.39	4.87
Age	1.30	.109	0.94	1.79

*note. Wave 2 sampling weights used
